# Population History and Natural Selection Shape Patterns of Genetic Variation in 132 Genes

**DOI:** 10.1371/journal.pbio.0020286

**Published:** 2004-09-07

**Authors:** Joshua M Akey, Michael A Eberle, Mark J Rieder, Christopher S Carlson, Mark D Shriver, Deborah A Nickerson, Leonid Kruglyak

**Affiliations:** **1**Division of Human Biology, Fred Hutchinson Cancer Research CenterSeattle, WashingtonUnited States of America; **2**Department of Genome Sciences, University of WashingtonSeattle, WashingtonUnited States of America; **3**Department of Anthropology, Pennsylvania State UniversityUniversity Park, PennsylvaniaUnited States of America; **4**Howard Hughes Medical Institute, Chevy ChaseMarylandUnited States of America

## Abstract

Identifying regions of the human genome that have been targets of natural selection will provide important insights into human evolutionary history and may facilitate the identification of complex disease genes. Although the signature that natural selection imparts on DNA sequence variation is difficult to disentangle from the effects of neutral processes such as population demographic history, selective and demographic forces can be distinguished by analyzing multiple loci dispersed throughout the genome. We studied the molecular evolution of 132 genes by comprehensively resequencing them in 24 African-Americans and 23 European-Americans. We developed a rigorous computational approach for taking into account multiple hypothesis tests and demographic history and found that while many apparent selective events can instead be explained by demography, there is also strong evidence for positive or balancing selection at eight genes in the European-American population, but none in the African-American population. Our results suggest that the migration of modern humans out of Africa into new environments was accompanied by genetic adaptations to emergent selective forces. In addition, a region containing four contiguous genes on Chromosome 7 showed striking evidence of a recent selective sweep in European-Americans. More generally, our results have important implications for mapping genes underlying complex human diseases.

## Abstract 2

An analysis of 132 human genes suggests that the migration of modern humans out of Africa into new environments was accompanied by genetic adaptations to emergent selective forces

## **Introduction**

Despite intense study and interest, a detailed understanding of the evolutionary and demographic forces that have shaped extant patterns of human genomic variation remains elusive. An important goal in studies of DNA sequence variation is to identify loci that have been targets of natural selection and thus contribute to differences in fitness between individuals in a population. Identifying regions of the human genome that have been subject to natural selection will provide important insights into recent human history (Sabeti et al. 2002; Tishkoff and Verrelli 2003), the function of genes (Akey et al. 2002), and the mechanisms of evolutionary change (Otto 2000), and it may also facilitate the identification of complex disease genes (Jorde et al. 2001; Nielsen 2001).

The neutral theory of molecular evolution (Kimura 1968; King and Jukes 1969), which posits that the majority of polymorphisms have no appreciable effects on fitness, has been integral to recent studies of natural selection. Specifically, the neutral theory makes explicit and quantitative predictions about the amount, structure, and patterns of sequence variation expected under neutrality, and serves as a null hypothesis by which to evaluate the evidence for or against selection in empirical data (Otto 2000; Nielsen 2001). Unfortunately, robust inferences of natural selection from DNA sequence data are difficult because of the confounding effects of population demographic history. For example, both positive selection and increases in population size lead to an excess of low-frequency alleles in a population relative to what is expected under a standard neutral model (i.e., a constant-size, randomly mating population at mutation-drift equilibrium). Therefore, rejection of the standard neutral model usually cannot be interpreted as unambiguous evidence for selection.

One way out of this conundrum is to recognize that population demographic history affects patterns of variation at all loci in a genome in a similar manner, whereas natural selection acts upon specific loci (Cavalli-Sforza 1966; Przeworski et al. 2000; Andolfatto 2001; Nielsen 2001). Thus, by sampling a large number of unlinked loci throughout the genome, it is in principle possible to distinguish between selection and demography. For instance, Akey et al. (2002) recently used this approach to infer the presence of selection in a genome-wide collection of single nucleotide polymorphisms (SNPs). However, studies based on SNPs that were initially identified in a small sample and subsequently genotyped in a larger sample are not ideally suited for detecting selection, because ascertainment bias (i.e., a systematic bias introduced into a dataset because of the way in which the data were collected) complicates downstream analyses (Akey et al. 2003). However, DNA sequence data provides the opportunity to exhaustively catalog variation, which attenuates the problem of ascertainment bias and therefore is arguably the most powerful and direct approach for detecting selection.

Here, we describe an extensive analysis of the molecular evolution of 132 genes that were comprehensively resequenced in 24 African-Americans and 23 European-Americans. In total, over 2.5 Mb of baseline reference DNA was sequenced, spanning 20 autosomal chromosomes and the X chromosome. The sampling of a large number of loci dispersed throughout the genome has allowed us to clarify the relative contributions of demography and selection to patterns of genetic variation at individual genes. Specifically, we developed a rigorous computational approach for taking into account multiple hypothesis tests and demographic history, and we found that while many apparent selective events can instead be explained by demography, there is also strong evidence for positive or balancing selection at eight genes in the European-derived population. In addition, we describe a striking example of a previously unreported recent selective sweep in European-Americans that spans four contiguous genes on Chromosome 7. More generally, our data provide insight into the demographic histories of African-American and European-American populations and have important implications for genetic association studies of complex diseases, as several of the genes showing evidence of selection have been implicated in susceptibility to complex human diseases.

## **Results**

### Statistical Tests Reveal Many Deviations from Neutrality

**Figure 1:**
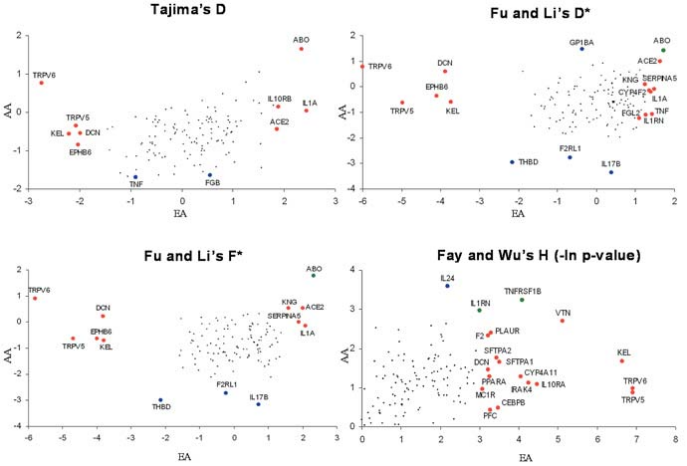
Scatter Plot of Neutrality Test Statistics in European- and African-Americans Genes that are nominally significant (*p* < 0.05) in European-Americans (EA), African-Americans (AA), or both populations are denoted by red, blue, and green circles, respectively. Genes that are not significant are shown as black dots. Two-sided tests were used for Tajima's D, Fu and Li's D*, and Fu and Li's F*, and a one-sided test was used for Fay and Wu's H.

We resequenced 132 genes primarily involved in inflammation, blood clotting, and blood pressure regulation and discovered a total of 12,890 SNPs (Table S1). We first characterized patterns of genetic variation by calculating several common summary statistics of the within-population allele frequency distribution, including Tajima's D, Fu and Li's D*, Fu and Li's F*, and Fay and Wu's H. As is conventionally done, we initially determined whether these statistics were significantly different from what is expected under a standard neutral model by performing coalescent simulations under the simplifying assumption of no recombination. In total, 28 genes in the European-American sample and ten genes in the African-American sample were nominally significant (i.e., the observed test statistic differed from neutral expectations at *p* < 0.05) in one or more tests of the allele frequency distribution (Figure 1). Thus, the European-American sample contained nearly three times as many significant genes as the African-American sample, and only three genes were significant in both samples *(ABO, IL1RN,* and *TNFRSF1B)*.

The direction of Tajima's D, Fu and Li's D*, and Fu and Li's F* is potentially informative about the evolutionary and demographic forces that a population has experienced. For example, negative values reflect an excess of rare polymorphisms in a population, which is consistent with either positive selection or an increase in population size. Positive values indicate an excess of intermediate-frequency alleles in a population and can result from either balancing selection or population bottlenecks. In the European-American sample, we observed eleven significantly positive and five significantly negative values for one or more of these three test statistics (Figure 1). In the African-American sample, we observed two significantly positive and five significantly negative values for one or more of the test statistics (Figure 1).

The observations of both significantly positive and significantly negative values of Tajima's D, Fu and Li's D*, and Fu and Li's F*, combined with the largely nonoverlapping set of significant genes, could reflect selective pressures unique to one population (i.e., local adaptation), different demographic histories, spurious results, or most likely some complex combination of all of these factors. Although these results are intriguing, their interpretation is confounded by two issues: (1) We have not corrected for multiple hypothesis tests, and (2) rejection of the standard neutral model can result from either selective or demographic forces. In the subsequent sections, we develop approaches to address these issues with the dual goals of identifying genes that possess strong evidence of natural selection and of inferring population demographic history.

### Correcting for Multiple Hypothesis Tests

In order to robustly correct for multiple hypothesis tests, the conventional practice of assuming no recombination when determining significance is not appropriate, because it results in conservative *p* values (Wall 1999) and hence decreases the statistical power to detect deviations from neutrality. Although recombination can easily be incorporated into coalescent simulations, in practice it is difficult to accurately estimate recombination rates, which vary substantially across the genome (Yu et al. 2001; McVean et al. 2004). To model the stochastic behavior and uncertainty in local rates of recombination, we reassessed the significance of Tajima's D, Fu and Li's D*, Fu and Li's F*, and Fay and Wu's H by coalescent simulations that incorporate recombination rates sampled from a Gamma(2, 0.5 × 10^–8^) distribution (see Materials and Methods). Finally, we corrected each statistic for multiple tests using the positive false discovery rate (FDR; Storey 2002) method, which determines the predicted proportion of “false positives” for the number of significant observations.

In the European-American sample, we observed 22 genes that were significant at a FDR of 5% (i.e., we expect approximately one false positive in this set of genes) for one or more tests of the allele frequency distribution (Tables 1 and S2). Thus, the number of significant genes in the European-American sample, after incorporating recombination and correcting for multiple tests, is very similar to the initial results where recombination was ignored and multiple tests were not corrected for. However, in the African-American sample there were no genes significant at a FDR of 5% for any of the tests of the allele frequency distribution (unpublished data). This result is consistent with the relatively small number of significant genes that were initially found before correcting for multiple tests (Figure 1). Genes with the smallest FDR in African-Americans were *ABO, F2RL1,* and *IL17B,* which each had a FDR of 13.5% for Fu and Li's D*.

**Table 1:**
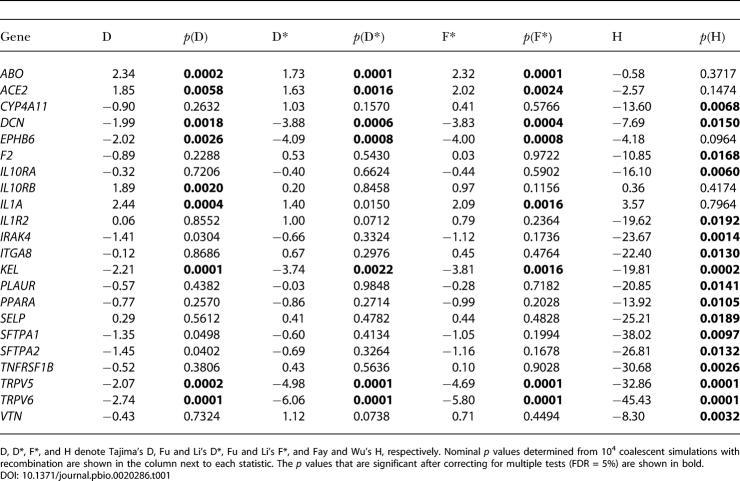
Significant Genes in European-Americans after Correcting for Multiple Tests

D, D*, F*, and H denote Tajima's D, Fu and Li's D*, Fu and Li's F*, and Fay and Wu's H, respectively. Nominal *p* values determined from 10^4^ coalescent simulations with recombination are shown in the column next to each statistic. The *p* values that are significant after correcting for multiple tests (FDR = 5%) are shown in bold

### Distinguishing between Selective and Demographic Forces

Although neutrality tests of the allele frequency distribution reveal many significant deviations, it is impossible to unambiguously interpret these data as evidence for natural selection, because the null model used to assess significance makes unrealistic assumptions about population demographic history. In principle, it is possible to distinguish between demography and selection, because demography affects all loci in the genome, whereas selection acts upon specific loci. Thus, by sampling a large number of loci dispersed throughout the genome, we can begin to construct a more realistic null hypothesis by which to evaluate the evidence for or against selection (Kreitman 2000).

**Figure 2:**
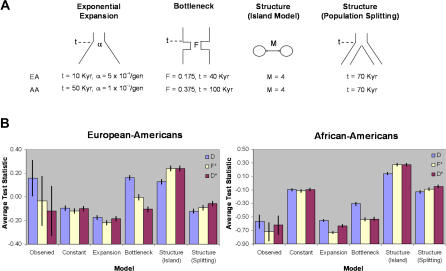
Summary of the Four Demographic Models Considered in Each Population (A) Schematic diagram of each demographic model and its associated parameters (see Materials and Methods for details). Parameter values that match the observed data most closely for European-Americans (EA) and African-Americans (AA) are shown below the diagrams. (B) Average and 95% confidence intervals of Tajima's D (blue bars), Fu and Li's D* (red bars), and Fu and Li's F* (pale yellow bars) for the observed data and each demographic model (using the parameters that most closely match the empirical data). Results from the standard neutral model (Constant) are also shown.

To this end, we used the empirical data to explore four different demographic models (Figure 2A), which we could then use to account for demographic influences on tests of natural selection. For each model, we used coalescent theory to simulate data over a broad range of parameters and identified the particular combination of parameters that most closely matched summary statistics (average Tajima's D, Fu and Li's D*, and Fu and Li's F*) of the observed data. Of the four demographic models, the European-American data are most consistent with a bottleneck occurring approximately 40,000 y ago, which is nearly identical to a previously reported estimate (Sabeti et al. 2002). However, the confidence intervals for the observed summary statistics are broad, and various aspects of the data are also consistent with other models (Figure 2B). The African-American data are most consistent with either an exponential expansion or a relatively old and severe bottleneck (Figure 2). Similarly, using DNA sequence variation from ten unlinked, noncoding loci, Pluzhnikov et al. (2002) found that an African Hausa sample was consistent with a recent population expansion (although they did not consider bottleneck models).

**Figure 3:**
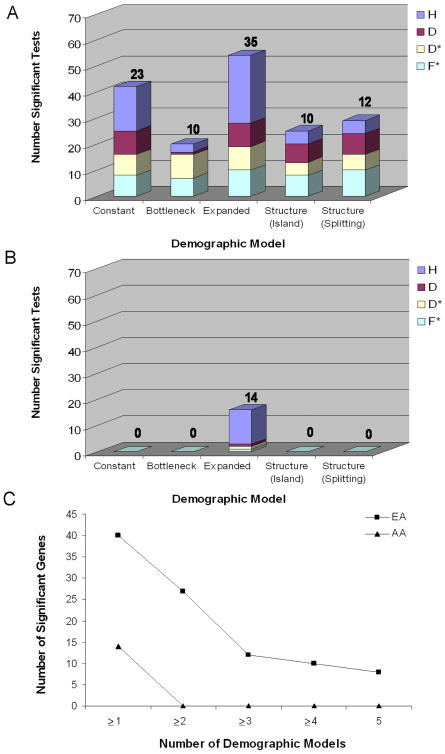
The Influence of Demographic History on Tests of Selection (A and B) The significance of observed values of Tajima's D (red), Fu and Li's D* (pale yellow), Fu and Li's F* (pale blue), and Fay and Wu's H (dark blue) were reassessed for each best-fit demographic model in European-Americans (A) and African-Americans (B). Results from the standard neutral model (Constant) are shown for comparison. The number of significant genes for each demographic model is noted above each category in (A) and (B). For example, there were a total of 19 significant test statistics across all four tests of neutrality assuming a bottleneck model for Europeans, which define ten unique genes. Therefore, each gene is supported by approximately two (19/10) tests of neutrality. (C) The distribution of the number of significant genes across the five demographic models in European-Americans and African-Americans. For example, in European-Americans, 40 genes were significant in at least one of the demographic models, and 27 genes were significant in at least two of the demographic models.

We reestimated the significance of Tajima's D, Fu and Li's D*, Fu and Li's F*, and Fay and Wu's H in each population for each of the four demographic models using the best-fit parameter values. All simulations included recombination and correction for multiple tests using the FDR method (with a FDR of 5%) as described above. Population history can clearly have a profound effect on tests of natural selection (Figure 3A and 3B; see also Simonsen 1995; Przeworski 2002), and given the uncertainty in our knowledge of human demographic history, it is challenging to ascribe unusual patterns of genetic variation to either demography or selection. To address this problem, we identified genes whose statistical evidence for selection was robust to demographic history. We conservatively defined demographically robust selection genes as those that demonstrated significant evidence for selection in all five demographic models. We identified eight demographically robust selection genes in European-Americans, and zero in African-Americans (Figure 3C; Table 2). Thus, out of the 22 genes originally found to be significant (at a FDR of 5%) under a standard neutral model, our estimates suggest that demographic history can potentially account for approximately two-thirds of these observations.

**Table 2:**
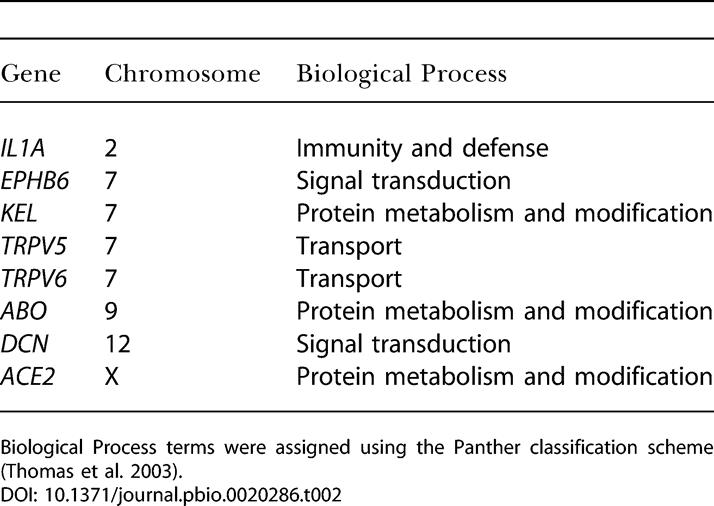
Demographically Robust Selection Genes in European-Americans

Biological Process terms were assigned using the Panther classification scheme (Thomas et al. 2003)

### Evidence for a Recent Selective Sweep on Chromosome 7q in European-Americans

**Figure 4:**
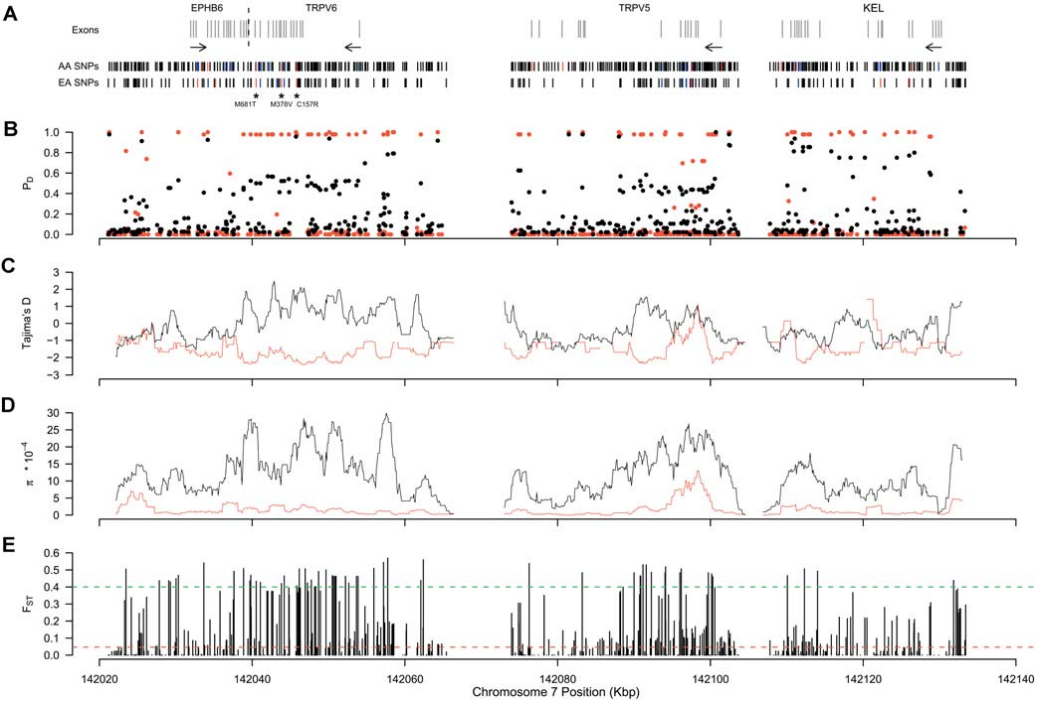
A Strong Signature of Positive Selection Spanning 115 kb on Chromosome 7q (A–D) Exons for *EPHB6, TRPV6, TRPV5,* and *KEL* are shown as gray vertical lines. A dashed black line indicates the boundary between *EPHB6* and *TRPV6* exons, which are approximately 1 kb apart. Transcriptional orientation is indicated by the arrows below exon positions. SNPs found in European-Americans and African-Americans are shown below. Noncoding, synonymous, and nonsynonymous SNPs are denoted as black, blue, and red vertical bars, respectively. The positions of three nonsynonymous SNPs in *TRPV6* are shown with asterisks. For each of the resulting nonsynonymous amino acid changes, the most frequent amino acid in European-Americans is given first. The frequency of derived alleles, P_D_ (B), sliding window plots of Tajima's D (C), and nucleotide diversity, π (D), are shown across the entire region. Gaps in the sliding window plots indicate positions where sequence data were not obtained. In (B–D), European- and African-American data are shown in red and black, respectively. (E) The distribution of F_ST_ across the 115-kb region. The average F_ST_ for all SNPs across the 132 genes is shown as a dashed red line. The dashed green line indicates the threshold for significantly (*p* < 0.01) large values of F_ST_, determined by coalescent simulations.

One particularly interesting region of the genome is located at 7q and contains four contiguous demographically robust selection genes (*EPHB6, TRPV6, TRPV5,* and *KEL;* Figure 4A). Collectively, the entire 115-kb region bears many of the hallmarks of a locus subject to a recent selective sweep: an excess of high-frequency-derived alleles (Figure 4B); an overall excess of rare polymorphisms, which results in an extreme skew of the site frequency spectrum reflected by sharply negative values of Tajima's D (Figure 4C); and a significant reduction in the amount of nucleotide diversity (Figure 4D). The signature of positive selection is seen only in European-Americans, suggesting that *EPHB6, TRPV6, TRPV5,* and/or *KEL* possess specific alleles that have conferred local adaptation to a unique environmental pressure in European-derived populations. Consistent with this hypothesis, we observed strong levels of population subdivision (Figure 4E) across the entire 115-kb region. The closest genes centromeric to *EPHB6* and telomeric to *KEL* are approximately 42 kb and 64 kb away, respectively, suggesting that one or more of these four genes is the target of selection. However, we have not surveyed patterns of DNA sequence variation outside of the region delimited by *EPHB6* and *KEL,* and therefore it is possible that the signature of selection extends even further. Based on the level of genetic variation on the putatively selected haplotype (see Materials and Methods), we can provide a rough estimate of the time back to the selective sweep as approximately 10,000 y ago. Although this number should be interpreted cautiously, it suggests that selection operated recently.

## **Discussion**

In summary, we have found that both population demographic history and natural selection shaped patterns of DNA sequence variation in the 132 genes studied here. By studying multiple unlinked loci dispersed throughout the genome, we were able to develop a rigorous computational approach to distinguish between the confounding effects of natural selection and demographic history on patterns of genetic variation. Using this strategy, we found that approximately two-thirds of the genes that were initially significant could be accounted for by population demographic history. Thus, our analyses clearly demonstrate the importance of considering both neutral and nonneutral forces when interpreting DNA sequence variation.

An interesting feature of our data is that the majority of deviations from neutrality, and all of the demographically robust selection genes, are not shared between the two population samples, suggesting that local adaptation has played an important role in recent human evolutionary history. Consistent with this observation, several possible examples of local adaptation in humans have previously been reported (Stephens et al. 1998; Rana et al. 1999; Hollox et al. 2001; Tishkoff et al. 2001; Currat et al. 2002; Fullerton et al. 2002; Gilad et al. 2002; Hamblin et al. 2002; Rockman et al. 2003). We hypothesize that the stronger signature of selection in the European-derived population may reflect the exposure of non-African populations to novel and evolutionarily recent selective pressures (e.g., unique dietary, climatic, and cultural environments) as modern humans migrated out of Africa and spread throughout the world. In contrast, the African-derived population may have experienced fewer evolutionarily recent selective forces. Theoretical studies have demonstrated that the power to detect a selective sweep is generally greatest if it occurred less than approximately 0.1 N_e_ generations ago (i.e., approximately 20,000–25,000 y ago [Kim and Stephan 2000; Przeworski 2002]), which is consistent with our hypothesis that signatures of selection in European-Americans reflect recent selective events. However, it is important to note that we have surveyed less than 1% of all human genes, and many of the genes that we did analyze are involved in mediating inflammatory and immune responses; thus our results may not be representative of the genome at large. Interestingly, Glinka et al. (2003) found that European-derived populations of Drosophila melanogaster demonstrated abundant evidence for recent selective sweeps, whereas African populations did not, which is strikingly similar to our results in humans.

An alternative explanation for why we observed fewer significant results in African-Americans than in European-Americans is that African-Americans are an admixed population (Parra et al. 1998), and the admixture process may mask the signature of selection. However, simulation studies in which we constructed an artificially admixed European-American sample with African-American chromosomes resulted in an increase in significant genes relative to the observed data (unpublished data). Therefore, to the extent that our simulations recapitulate the dynamics of the admixture process in African-Americans, admixture is unlikely to explain the discrepancies between the two samples.

It is important to point out that some genes that do not meet our rigorous definition of a high-confidence selection gene may have nonetheless been targets of selection, such as *ABO* in African-Americans (Table S2). In this initial survey we have elected to be conservative and identify genes that possess the strongest signatures of selection. Ultimately, it will be necessary to confirm our results in geographically diverse populations (a more comprehensive sampling of African populations is particularly needed), as well as in replicate samples of the populations we studied, and to functionally characterize the suspected targets of selection.

Recently, Clark et al. (2003) presented an evolutionary analysis of 7,645 orthologous human-chimp-mouse gene trios by looking for accelerated rates of synonymous and nonsynonymous nucleotide substitution in either the human or the chimp lineages. In total, 50 genes overlap between our dataset and theirs (Table S3), including three demographically robust selection genes (*TRPV6, EPHB6,* and *DCN;* see Table 2). All three of the demographically robust selection genes also demonstrate statistically significant evidence (*p* < 0.05) of accelerated evolution in either the human *(TRPV6* and *EPHB6)* or chimp *(DCN)* lineage. In addition, Clark et al. (2003) found evidence for accelerated evolution in seven genes along the human lineage that did not demonstrate evidence for selection in our dataset (Table S3). This observation may simply reflect either false negatives in our analysis or false positives in Clark et al. (2003). However, it is important to note that the statistical methods and data used to detect selection in Clark et al. (2003) (divergence between species) are quite different from our methods (polymorphism within species), so completely overlapping results are not expected. More specifically, the analyses of Clark et al. (2003) will preferentially detect selective events between species, whereas our analyses will preferentially identify selection operating within species. In other words, these two methods are complimentary and may potentially detect selection operating over different time scales. In this respect, it is particularly interesting that the genes we identified as possessing the strongest evidence for recent selection in one human population also show evidence of selection in the human or chimp lineage following their divergence (Clark et al. 2003).

The strongest signature of selection that we observed occurs on Chromosome 7q in European-Americans. The signature of selection extends for at least 115 kb and spans the genes *EPHB6, TRPV6, TRPV5,* and *KEL*. To our knowledge, this is the largest footprint of selection that has been described in the human genome, and likely reflects the combination of strong and recent selective pressures and reduced recombination in this region (the average ratio of genetic to physical distance, cM/Mb, is approximately 0.68 according to the deCode map). Based on our current data it is impossible to identify which gene (or perhaps genes) has been the target (or targets) of selection. However, *TRPV6* is a particularly interesting candidate, as it possesses three nonsynonymous amino acid substitutions (C157R, M378V, and M681T) that are each nearly fixed for the derived allele in European-Americans, show significant frequency differences between European-Americans and African-Americans, and are located in the most significant regions of both Tajima's D and reduced nucleotide diversity (Figure 4). The program PolyPhen (Ramensky et al. 2002) predicts that the C157R replacement may alter protein structure. Recently, TRPV6 was shown to be up-regulated in prostate cancer (Wissenbach et al. 2001), and a susceptibility locus for aggressive prostate cancer was mapped to the *TRPV6* region (7q31–33; Paiss et al. 2003). These observations, combined with the large difference in disease prevalence between Europeans and African-Americans (Crawford 2003), make *TRPV6* a strong candidate gene for prostate cancer susceptibility and/or aggressiveness.

*TRPV6,* as well as *TRPV5,* constitute the rate-limiting step in kidney, intestine, and placenta calcium absorption (Nijenhuis et al 2003; van de Graaf et al. 2003). Interestingly, Northern European populations have very high frequencies of the lactase persistence allele (LCT*P; Hollox et al. 2001), which allows digestion of fresh milk throughout adulthood. It is widely accepted that strong selection has driven LCT*P to high frequency in Northern Europeans, beginning sometime after the domestication of animals approximately 9,000 y ago (Feldman and Cavalli-Sforza 1989; Hollox et al. 2001; Bersaglieri et al. 2004). What has been debated, however, is the specific selective advantage conferred by lactase persistence (Holden and Mace 1997). Our finding that *TRPV6* and/or *TRPV5* have been under strong selective pressure in Northern Europeans suggests that increased calcium absorption may have been the driving force behind selection for lactase persistence, which was originally hypothesized by Flatz and Rotthauwe (1973). Although additional studies are clearly needed, our results provide additional insight into the molecular mechanisms of adaptation to a new dietary niche (i.e., high-lactose diets).

More generally, our results have several implications for mapping genes underlying complex human diseases. Specifically, four of the high-confidence selection genes have been implicated in various complex diseases (Table 3). If genes underlying complex diseases have experienced differential selective pressures, then this could in part explain the failure of many studies to replicate disease associations across populations (Florez et al. 2003; Moore 2003). Finally, our data are consistent with the notion that variation in genes that was once beneficial may have become detrimental in the environmental and cultural milieu of contemporary human populations, akin to the “thrifty gene” hypothesis for type II diabetes (Neel 1962).

**Table 3:**
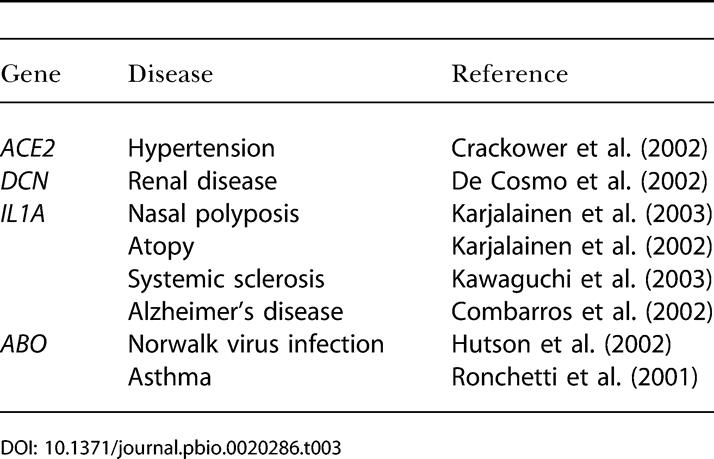
Disease Associations with Demographically Robust Selection Genes

## Materials and Methods

### 

#### **DNA samples and sequencing**

Human DNAs were obtained from the Coriell Institute (Camden, New Jersey, United States). We analyzed DNA from 24 African-Americans from the Human Variation Panel, African-American Panel of 50 (HD50AA) and DNA from 23 European-Americans derived from various CEPH pedigrees. We also sequenced each gene in a common chimpanzee (Pan troglodytes) to determine the derived allele for Fay and Wu's H test. These data were generated under the auspices of the SeattleSNPs Program for Genomic Applications, which resequences candidate genes involved in inflammatory processes in humans. In general, we resequenced the complete genomic region for each gene, including introns and approximately 2 kb 5′ of the gene and 1 kb 3′ of the gene using Big-Dye terminator chemistry on an ABI 3700 or ABI 3730XL (Applied Biosystems, Foster City, California, United States). For several exceptionally large genes, such as *F13A1,* less than complete coverage was obtained (see Table S1). All variants occurring once in the sample were confirmed with an additional sequencing run. Further experimental details and all of the raw data can be found at our website (http://pga.gs.washington.edu/).

#### **Data analysis**

We calculated the following summary statistics of nucleotide variation for each gene: θ^= *S/a_n_,* where *S* is the number of segregating sites, 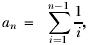 and *n* is the sample size (Watterson 1975); 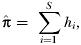 , where *h_i_* is an unbiased estimate of nucleotide diversity for the *i*th segregating site (see equation 12 in Tajima 1989) and *η_S_*, which is the number of singletons (Fu and Li 1993). From these statistics we calculated several tests of the standard neutral model including Tajima's D (Tajima 1989), Fu and Li's D* (Fu and Li 1993), Fu and Li's F* (Fu and Li 1993), and Fay and Wu's H statistic (Fay and Wu 2000). In calculating Fu and Li's F*, we used the formulas provided in Simonsen et al. (1995), which correct a typographical error in the original description of the method (Fu and Li 1993). For a discussion of the similarities and differences of Tajima's D, Fu and Li's D*, Fu and Li's F*, and Fay and Wu's H, see Fu and Li (1993), Simonsen et al. (1995), and Przeworski (2002).

We initially assessed the significance of these statistics by comparing the observed values to 10^4^ coalescent simulations (Hudson 1983), conditional on the observed sample size and number of segregating sites, assuming a standard neutral model with no recombination. Coalescent simulations were performed using the program ms (obtained from R. Hudson's Web site [http://home.uchicago.edu/~rhudson1/source.html]). In order to correct for multiple tests, we repeated the coalescent simulations as described above, but included recombination. Following Pluzhnikov et al. (2002), for each of the 10^4^ coalescent realizations, we sampled the recombination rate from a Gamma(2, 0.5 × 10^–8^) distribution whose expectation equals the average genome-wide recombination rate of 10^–8^/generation (Hamblin et al. 2002). The positive FDR method was used to correct for multiple hypothesis tests using the software QVALUE (Storey 2002; http://faculty.washington.edu/~jstorey/qvalue/).

We quantified the allele frequency differences between the European- and African-American samples by the statistic F_ST_ as described in Akey et al. (2002). All of the analyses described above excluded insertion/deletion polymorphisms, but their inclusion does not affect any of our conclusions (unpublished data). We assigned PANTHER Biological Process terms (Thomas et al. 2003) to each gene.

We estimated the time since the selective sweep for the Chromosome 7q region in European-Americans by analyzing the amount of nucleotide diversity that has accumulated on the selected haplotype as described in Rozas et al. (2001). We assumed that *TRPV6* is the target of selection and the selected haplotype is defined by the C157R, M378V, and M681T polymorphisms. If mutations are Poisson-distributed, the expected number of segregating sites in a genealogy is *E*[*S*] = *μE*[*T*], where *S, μ,* and *T* denote segregating sites, neutral mutation rate of the locus, and total branch length of the genealogy, respectively. Assuming a star-shaped genealogy, *E*[*T*] = *n* × *t*, where *n* is the number of selected haplotypes. Thus, the time back to the selective sweep, *t,* can be estimated by *S*/(*nμ*). For *TRPV6* in European-Americans, *n* = 45 (i.e., 45 out of 46 haplotypes carry C157, M378, and M681), *S* = 11, and *μ* = 2.5 × 10^–5^.

#### **Demographic modeling**

We assessed the impact of demographic history on the robustness of the statistical tests of neutrality by using coalescent theory to simulate data under four different population histories, including a bottleneck, exponential expansion, population structure according to an island model that allows symmetric migration between demes, and population structure assuming population splitting with no subsequent migration. For each model we simulated data under a wide variety of parameters by conditioning on the observed sample size and θ^_W_ for each population. The bottleneck model is specified by the parameters *F* (the inbreeding coefficient) and *t* (the time in years measured from the present) at which the bottleneck occurred. Values of *F* and *t* considered were *F* = [0.05, 0.075, … , 0.40] and *t* = [10,000, 20,000, … , 100,000]. The exponential expansion model is determined by the parameters *α* (the growth rate/generation) and *t* (the time, in years measured from the present, at which the population began increasing in size). Values considered for *α* and *t* were: *α* = [0.0005, 0.001, … , 0.01] and *t* = [10,000, 20,000, … , 100,000]. The population structure under an island model is specified by the population migration rate between demes, *M* = 4*N_o_m,* where *N_o_* and *m* are the effective subpopulation size and fraction of migrants in each subpopulation per generation, respectively. Values of *M* considered were *M* = [1, 2, … , 10]. The structure model assuming population splitting with no subsequent migration is determined by the parameter *t* (the time in years since the populations diverged). Values of *t* considered were *t* = [1,000, 2,000, …, 10,000]. In all simulations we assumed an effective population size of 10,000 and a generation time of 25 y in order to facilitate comparisons to a previous study (Sabeti et al. 2002). The parameter space for each model included a full grid search, so we tested 160, 100, 10, and 10 parameter combinations for the bottleneck, expansion, structure (island), and structure (splitting) models, respectively. We performed 10^4^ simulations for each parameter combination.

For each demographic model, we calculated the average value of Tajima's D, Fu and Li's D*, and Fu and Li's F* and compared the results to the observed values of these statistics. For the bottleneck and exponential expansion models, we identified the parameter values that most closely matched the observed data by identifying the parameter combination that minimized the function 
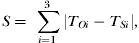
 , where *T_Oi_* and *T_Si_* denote the observed and simulated averages of Tajima's D, Fu and Li's D*, and Fu and Li's F*. For the demographic models of population structure we selected parameter values that matched the observed F_ST_. Finally, we reassessed the significance of the observed values of Tajima's D, Fu and Li's D*, Fu and Li's F*, and Fay and Wu's H by 10^4^ coalescent simulations for each demographic model using the best-fit parameter values.

## Supporting Information

Table S1 Summary Statistics of the 132 Genes (266 KB DOC).

Table S2 Neutrality Test Statistics (534 KB DOC).

Table S3 Overlap of Genes Analyzed by Clark et al. (2003)
(87 KB DOC).

### Accession Numbers

LocusLink ID numbers (http://www.ncbi.nlm.nih.gov/LocusLink/) for the genes discussed in this paper are *ABO* (28), *ACE2* (59272), *APOH* (350), *BDKRB2* (624), *BF* (629), *C2* (717), *CCR2* (1231), *CD36* (948), *CEBPB* (1051), *CRF* (10882), *CRP* (1401), *CSF2* (1437), *CSF3* (1440), *CSF3R* (1441), *CYP4A11* (1579), *CYP4F2* (8529), *DCN* (1634), *EPHB6* (2051), *F10* (2159), *F11* (2160), *F12* (2161), *F13A1* (2162), *F2* (2147), *F2R* (2149), *F2RL1* (2150), *F2RL2* (2151), *F2RL3* (9002), *F3* (2152), *F5* (2153), *F7* (2155), *F9* (2158), *FGA* (2243), *FGB* (2244), *FGG* (2266), *FGL2* (10875), *FSBP* (10646), *GP1BA* (2811), *ICAM1* (3383), *IFNG* (3458), *IGF2* (3481), *IGF2AS* (51214), *IL10* (3586), *IL10RA* (3587), *IL10RB* (3588), *IL11* (3589), *IL12A* (3592), *IL12B* (3593), *IL13* (3596), *IL15RA* (3601), *IL17B* (27190), *IL19* (29949), *IL1A* (3552), *IL1B* (3553), *IL1R1* (3554), *IL1R2* (7850), *IL1RN* (3557), *IL2* (3558), *IL20* (50604), *IL21R* (50615), *IL22* (50616), *IL24* (11009), *IL2RB* (3560), *IL3* (3562), *IL4* (3565), *IL4R* (3566), *IL5* (3567), *IL6* (3569), *IL8* (3576), *IL9* (3578), *IL9R* (3581), *IRAK4* (51135), *ITGA2* (3673), *ITGA8* (8516), *JAK3* (3718), *KEL* (3792), *KLK1* (3816), *KLKB1* (3818), *KNG* (3827), *LTA* (4049), *LTB* (4050), *MAP3K8* (1326), *MC1R* (4157), *MMP3* (4314), *MMP9* (4318), *NOS3* (4846), *PFC* (5199), *PLAT* (5327), *PLAU* (5328), *PLAUR* (5329), *PLG* (5340), *PON1* (5444), *PON2* (5445), *PPARA* (5465), *PPARG* (5468), *PROC* (5624), *PROCR* (10544), *PROS1* (5627), *PROZ* (8858), *PTGS2* (5743), *SCYA2* (6347), *SELE* (6401), *SELL* (6402), *SELP* (6403), *SELPLG* (6404), *SERPINA5* (5104), *SERPINC1* (462), *SERPINE1* (5054), *SFTPA1* (6435), *SFTPA2* (6436), *SFTPB* (6439), *SFTPC* (6440), *SFTPD* (6441), *SMP1* (23585), *STAT4* (6775), *STAT6* (6778), *TF* (7018), *TFPI* (7035), *TGFB3* (7043), *THBD* (7056), *TIRAP* (114609), *TNF* (7124), *TNFAIP1* (7126), *TNFAIP2* (7127), *TNFAIP3* (7126), *TNFRSF1A* (7132), *TNFRSF1B* (7133), *TRAF6* (7189), *TRPV5* (56302), *TRPV6* (55503), *VCAM1* (7412), *VEGF* (7422), and *VTN* (7448).

Coriell (http://coriell.undmj.edu/) repository numbers for human genomic DNAs sequenced for this study are as follows. DNAs from African-Americans were NA17101–NA17116 and NA17133–NA17140. DNAs from European-Americans were NA06990, NA07019, NA07348, NA07349, NA10830, NA10831, NA10842–NA10845, NA10848, NA10850–NA10854, NA10857, NA10858, NA10860, NA10861, NA12547, NA12548, and NA12560.

## Abbreviations

FDR: false discovery rate
SNP: single nucleotide polymorphism
