# Supplementary material for: Population History and Natural Selection Shape Patterns of Genetic Variation in 132 Genes
Source: PLoS Biol. 2004 Sep 7;2(10):e286. doi: 10.1371/journal.pbio.0020286 (PMC515367; doi:10.1371/journal.pbio.0020286)
Supplement: Table S1 — (266 KB DOC). [file pbio.0020286.st001.doc]

Supplementary Table 1. Summary Statistics of the 132 Genes.

| Gene | Chromosome | Seq. Length | Seq. Amount | Panther Biological Function | S | | π x 10^-4^ | |
| --- | --- | --- | --- | --- | --- | --- | --- | --- |
|  |  |  |  |  | EA | AA | EA | AA |
| *CYP4A11* | 1 | 14484 | 14235 | Lipid, fatty acid and steroid metabolism | 54 | 94 | 6.49 | 14.41 |
| *TNFRSF1B* | 1 | 45812 | 43118 | Immunity and defense | 117 | 159 | 5.38 | 5.83 |
| *IL24* | 1 | 11114 | 10628 | Immunity and defense | 19 | 36 | 5.24 | 6.47 |
| *CRP* | 1 | 6836 | 6715 | Immunity and defense | 12 | 27 | 4.28 | 8.39 |
| *CSF3R* | 1 | 18843 | 18843 | Immunity and defense | 50 | 96 | 5.66 | 9.27 |
| *F3* | 1 | 17222 | 16114 | Blood clotting; Immunity and defense | 24 | 56 | 4.99 | 6.00 |
| *F5* | 1 | 75774 | 54019 | Blood clotting; Immunity and defense | 202 | 304 | 9.50 | 11.75 |
| *IL10* | 1 | 7879 | 7879 | Immunity and defense | 24 | 26 | 9.66 | 9.32 |
| *IL19* | 1 | 10998 | 10998 | Signal transduction | 23 | 40 | 4.04 | 6.68 |
| *IL20* | 1 | 6634 | 6634 | Signal transduction | 17 | 25 | 4.73 | 6.91 |
| *PTGS2* | 1 | 12551 | 12551 | Immunity and defense | 30 | 48 | 4.16 | 6.57 |
| *SELE* | 1 | 14019 | 13892 | Cell adhesion | 64 | 81 | 8.95 | 10.26 |
| *SELL* | 1 | 24822 | 24304 | Cell adhesion | 95 | 145 | 7.64 | 12.10 |
| *SELP* | 1 | 44381 | 43454 | Cell adhesion | 133 | 243 | 7.56 | 9.89 |
| *SERPINC1* | 1 | 16330 | 15208 | Protein metabolism and modification; Immunity and defense | 26 | 39 | 3.13 | 5.50 |
| *SMP1* | 1 | 25269 | 23549 | Biological process unclassified | 34 | 81 | 3.24 | 6.83 |
| *VCAM1* | 1 | 22868 | 22868 | Signal transduction | 38 | 97 | 3.91 | 6.32 |
| *IL1A* | 2 | 18446 | 17849 | Immunity and defense | 47 | 70 | 10.27 | 9.03 |
| *IL1RN* | 2 | 19677 | 19677 | Immunity and defense | 89 | 138 | 12.81 | 10.94 |
| *IL1B* | 2 | 17447 | 17447 | Immunity and defense | 32 | 45 | 3.97 | 5.14 |
| *IL1R1* | 2 | 27864 | 27864 | Signal transduction | 82 | 129 | 6.85 | 7.51 |
| *IL1R2* | 2 | 23160 | 23160 | Signal transduction | 99 | 186 | 10.00 | 15.82 |
| *PROC* | 2 | 13870 | 12877 | Blood clotting; Immunity and defense | 38 | 51 | 9.86 | 8.92 |
| *SFTPB* | 2 | 11807 | 11094 | Blood circulation and gas exchange | 16 | 46 | 4.27 | 9.17 |
| *STAT4* | 2 | 52796 | 28360 | Signal transduction | 47 | 90 | 3.10 | 4.70 |
| *TFPI* | 2 | 38945 | 38209 | Blood clotting; Immunity and defense | 76 | 179 | 4.77 | 7.37 |
| *KNG* | 3 | 29388 | 27679 | Blood clotting; Immunity and defense | 146 | 189 | 17.12 | 19.89 |
| *PROS1* | 3 | 93089 | 54542 | Blood clotting | 42 | 72 | 1.69 | 1.61 |
| *CCR2* | 3 | 10073 | 10073 | Immunity and defense | 20 | 39 | 5.07 | 6.21 |
| *IL12A* | 3 | 11792 | 11330 | Immunity and defense | 28 | 49 | 7.60 | 9.13 |
| *PPARG* | 3 | 83894 | 40608 | Lipid, fatty acid and steroid metabolism | 97 | 128 | 6.37 | 6.84 |
| *TF* | 3 | 37308 | 36535 | Transport | 121 | 177 | 9.62 | 9.47 |
| *F11* | 4 | 27035 | 26603 | Blood clotting; Immunity and defense | 59 | 114 | 6.36 | 8.60 |
| *FGA* | 4 | 9946 | 9946 | Blood clotting; Immunity and defense | 17 | 23 | 3.18 | 3.99 |
| *FGB* | 4 | 11956 | 11604 | Blood clotting; Immunity and defense | 33 | 44 | 7.54 | 4.46 |
| *FGG* | 4 | 10168 | 10168 | Blood clotting; Immunity and defense | 12 | 21 | 2.28 | 3.49 |
| *IL2* | 4 | 6752 | 6752 | Immunity and defense | 9 | 18 | 3.51 | 3.08 |
| *IL8* | 4 | 7156 | 7035 | Immunity and defense | 8 | 31 | 4.05 | 5.70 |
| *KLKB1* | 4 | 33504 | 31670 | Blood clotting; Immunity and defense | 116 | 148 | 11.90 | 11.31 |
| *IL17B* | 5 | 9077 | 9077 | Immunity and defense | 22 | 31 | 7.28 | 4.81 |
| *F2RL1* | 5 | 18351 | 18351 | Blood clotting; Immunity and defense | 41 | 90 | 6.11 | 6.66 |
| *CSF2* | 5 | 5992 | 5992 | Immunity and defense | 17 | 29 | 6.68 | 10.77 |
| *F12* | 5 | 10616 | 10616 | Blood clotting; Immunity and defense | 21 | 43 | 3.99 | 6.08 |
| *F2R* | 5 | 24771 | 24231 | Blood clotting; Immunity and defense | 50 | 89 | 5.06 | 6.55 |
| *F2RL2* | 5 | 9273 | 9273 | Signal transduction | 34 | 55 | 10.70 | 12.42 |
| *IL12B* | 5 | 15648 | 14902 | Immunity and defense | 31 | 48 | 5.49 | 7.05 |
| *IL13* | 5 | 6919 | 6919 | Immunity and defense | 16 | 26 | 4.46 | 8.12 |
| *IL3* | 5 | 6387 | 6387 | Signal transduction | 9 | 27 | 3.07 | 5.47 |
| *IL4* | 5 | 22845 | 22845 | Immunity and defense | 52 | 95 | 4.53 | 8.70 |
| *IL5* | 5 | 5186 | 5186 | Immunity and defense | 3 | 15 | 0.92 | 4.37 |
| *IL9* | 5 | 6676 | 6676 | Immunity and defense | 12 | 27 | 3.38 | 6.22 |
| *ITGA2* | 5 | 105904 | 71521 | Cell adhesion | 258 | 317 | 9.21 | 10.93 |
| *TNF* | 6 | 4830 | 4830 | Immunity and defense | 11 | 21 | 3.63 | 4.71 |
| *F13A1* | 6 | 178952 | 29536 | Blood clotting; Immunity and defense | 121 | 185 | 12.08 | 13.23 |
| *BF* | 6 | 9956 | 9956 | Immunity and defense | 24 | 27 | 5.00 | 5.21 |
| *C2* | 6 | 21552 | 18626 | Immunity and defense | 36 | 51 | 3.61 | 4.52 |
| *LTA* | 6 | 5033 | 5033 | Immunity and defense | 19 | 19 | 11.48 | 9.37 |
| *LTB* | 6 | 4412 | 4412 | Immunity and defense | 7 | 15 | 2.07 | 3.75 |
| *PLG* | 6 | 55266 | 24465 | Protein metabolism and modification; Immunity and defense | 102 | 157 | 13.25 | 15.74 |
| *TNFAIP3* | 6 | 19810 | 19422 | Immunity and defense | 32 | 47 | 3.24 | 5.40 |
| *VEGF* | 6 | 15442 | 15442 | Signal transduction | 45 | 60 | 8.04 | 7.85 |
| *EPHB6* | 7 | 19655 | 19406 | Signal transduction | 37 | 93 | 1.83 | 8.45 |
| *KEL* | 7 | 25850 | 25850 | Protein metabolism and modification | 45 | 124 | 1.44 | 9.14 |
| *TRPV5* | 7 | 29923 | 29555 | Transport | 61 | 170 | 1.94 | 11.76 |
| *TRPV6* | 7 | 28272 | 27629 | Transport | 59 | 135 | 1.10 | 13.42 |
| *FGL2* | 7 | 6382 | 6382 | Blood clotting; Immunity and defense | 5 | 17 | 2.48 | 4.01 |
| *CD36* | 7 | 30180 | 29600 | Immunity and defense | 72 | 149 | 6.40 | 8.88 |
| *IL6* | 7 | 8019 | 7526 | Immunity and defense | 24 | 38 | 8.30 | 8.09 |
| *NOS3* | 7 | 25106 | 23307 | Signal transduction | 51 | 97 | 6.14 | 7.52 |
| *PON1* | 7 | 29370 | 29052 | Immunity and defense | 111 | 158 | 8.21 | 12.74 |
| *PON2* | 7 | 32705 | 32487 | Immunity and defense | 89 | 123 | 7.83 | 8.10 |
| *SERPINE1* | 7 | 14544 | 13208 | Protein metabolism and modification; Immunity and defense | 42 | 71 | 8.72 | 9.63 |
| *FSBP* | 8 | 9846 | 9846 | Blood clotting | 24 | 27 | 4.68 | 5.75 |
| *PLAT* | 8 | 36269 | 34026 | Blood clotting; Immunity and defense | 65 | 156 | 3.97 | 7.06 |
| *SFTPC* | 8 | 7746 | 7746 | Blood circulation and gas exchange | 30 | 37 | 10.32 | 9.08 |
| *ABO* | 9 | 23759 | 22302 | Protein metabolism and modification | 147 | 191 | 24.86 | 28.44 |
| *SFTPA1* | 10 | 24037 | 23126 | Immunity and defense | 143 | 159 | 8.90 | 13.96 |
| *SFTPA2* | 10 | 18039 | 18039 | Immunity and defense | 105 | 145 | 7.96 | 15.97 |
| *IL15RA* | 10 | 35809 | 35040 | Signal transduction | 134 | 207 | 9.75 | 11.18 |
| *ITGA8* | 10 | 206309 | 48348 | Cell adhesion | 113 | 241 | 5.17 | 9.62 |
| *MAP3K8* | 10 | 31602 | 31027 | Immunity and defense | 56 | 135 | 5.09 | 7.76 |
| *PLAU* | 10 | 10075 | 9274 | Blood clotting; Immunity and defense | 21 | 28 | 6.43 | 7.77 |
| *SFTPD* | 10 | 23538 | 23538 | Immunity and defense | 123 | 137 | 12.24 | 10.97 |
| *IL10RA* | 11 | 19942 | 19942 | Immunity and defense | 49 | 68 | 5.17 | 6.98 |
| *F2* | 11 | 22128 | 20407 | Blood clotting; Immunity and defense | 52 | 86 | 4.41 | 5.57 |
| *IGF2* | 11 | 9013 | 5251 | Signal transduction | 10 | 21 | 5.99 | 8.17 |
| *IGF2AS* | 11 | 20229 | 18382 | Biological process unclassified | 49 | 75 | 7.07 | 8.93 |
| *MMP3* | 11 | 13003 | 11903 | Protein metabolism and modification | 33 | 46 | 7.03 | 8.24 |
| *TIRAP* | 11 | 20684 | 20044 | Immunity and defense | 58 | 76 | 7.41 | 6.30 |
| *TRAF6* | 11 | 26556 | 23977 | Signal transduction | 47 | 87 | 3.10 | 5.56 |
| *DCN* | 12 | 35378 | 34947 | Signal transduction | 37 | 131 | 1.03 | 7.31 |
| *IRAK4* | 12 | 33033 | 33033 | Immunity and defense | 67 | 140 | 2.79 | 7.01 |
| *IFNG* | 12 | 7665 | 7665 | Immunity and defense | 12 | 28 | 4.01 | 4.87 |
| *IL22* | 12 | 8393 | 8393 | Immunity and defense | 28 | 45 | 9.62 | 9.99 |
| *SELPLG* | 12 | 16004 | 15817 | Immunity and defense | 47 | 75 | 7.18 | 8.73 |
| *STAT6* | 12 | 18891 | 18769 | Signal transduction | 21 | 50 | 2.86 | 4.13 |
| *TNFRSF1A* | 12 | 17350 | 16207 | Immunity and defense | 23 | 56 | 4.20 | 6.63 |
| *F10* | 13 | 29488 | 25406 | Blood clotting; Immunity and defense | 61 | 92 | 5.37 | 6.96 |
| *F7* | 13 | 18381 | 11924 | Blood clotting; Immunity and defense | 28 | 40 | 4.01 | 5.10 |
| *PROZ* | 13 | 16418 | 14366 | Blood clotting | 44 | 84 | 7.54 | 8.10 |
| *SERPINA5* | 14 | 8145 | 7806 | Protein metabolism and modification | 40 | 61 | 17.78 | 18.68 |
| *BDKRB2* | 14 | 15152 | 14050 | Signal transduction | 49 | 59 | 7.00 | 6.59 |
| *TGFB3* | 14 | 23990 | 23236 | Signal transduction | 48 | 79 | 4.60 | 6.85 |
| *TNFAIP2* | 14 | 15768 | 15644 | Immunity and defense | 46 | 66 | 6.55 | 8.77 |
| *MC1R* | 16 | 7046 | 6545 | Immunity and defense | 21 | 35 | 8.86 | 11.31 |
| *IL21R* | 16 | 26391 | 25844 | Immunity and defense | 68 | 107 | 6.54 | 7.59 |
| *IL4R* | 16 | 26550 | 25917 | Immunity and defense | 113 | 172 | 11.40 | 15.63 |
| *VTN* | 17 | 5559 | 5559 | Immunity and defense | 15 | 27 | 5.29 | 10.38 |
| *GP1BA* | 17 | 6241 | 6241 | Blood clotting | 21 | 26 | 7.76 | 8.08 |
| *CSF3* | 17 | 5527 | 5527 | Immunity and defense | 17 | 28 | 8.62 | 8.91 |
| *APOH* | 17 | 20299 | 17983 | Immunity and defense | 77 | 121 | 11.41 | 12.53 |
| *CRF* | 17 | 10451 | 9560 | Immunity and defense | 23 | 36 | 6.96 | 7.32 |
| *SCYA2* | 17 | 9174 | 9070 | Immunity and defense | 24 | 37 | 6.42 | 7.18 |
| *TNFAIP1* | 17 | 15259 | 14331 | Immunity and defense | 16 | 50 | 2.22 | 4.24 |
| *CYP4F2* | 19 | 23567 | 18701 | Lipid, fatty acid and steroid metabolism | 77 | 135 | 10.39 | 14.46 |
| *PLAUR* | 19 | 25423 | 23187 | Blood clotting; Immunity and defense | 92 | 159 | 7.63 | 11.94 |
| *F2RL3* | 19 | 11828 | 10214 | Signal transduction | 23 | 39 | 5.77 | 6.39 |
| *ICAM1* | 19 | 19022 | 17731 | Immunity and defense | 35 | 57 | 4.56 | 5.56 |
| *IL11* | 19 | 9803 | 8964 | Immunity and defense | 23 | 39 | 6.73 | 9.28 |
| *JAK3* | 19 | 21323 | 19067 | Signal transduction | 54 | 103 | 7.49 | 11.44 |
| *KLK1* | 19 | 10094 | 9922 | Protein metabolism and modification | 42 | 57 | 11.42 | 11.58 |
| *CEBPB* | 20 | 5086 | 4508 | Immunity and defense | 8 | 12 | 4.15 | 6.09 |
| *THBD* | 20 | 8532 | 7254 | Blood clotting; Immunity and defense | 12 | 22 | 2.48 | 3.31 |
| *MMP9* | 20 | 11224 | 11049 | Protein metabolism and modification | 26 | 45 | 6.30 | 8.70 |
| *PROCR* | 20 | 7199 | 6968 | Blood clotting; Immunity and defense | 13 | 14 | 5.20 | 4.77 |
| *IL10RB* | 21 | 36210 | 35918 | Immunity and defense | 118 | 202 | 11.43 | 13.30 |
| *PPARA* | 22 | 86215 | 35710 | Lipid, fatty acid and steroid metabolism | 78 | 163 | 3.92 | 8.04 |
| *IL2RB* | 22 | 26469 | 26029 | Signal transduction | 96 | 141 | 10.04 | 12.41 |
| *ACE2* | X | 41572 | 41262 | Protein metabolism and modification | 35 | 90 | 2.99 | 4.35 |
| *PFC* | X | 9760 | 9760 | Immunity and defense | 12 | 25 | 3.38 | 5.34 |
| *F9* | X | 35458 | 35458 | Blood clotting; Immunity and defense | 38 | 121 | 3.17 | 6.09 |
| *IL9R* | X | 15446 | 12523 | Immunity and defense | 55 | 94 | 9.17 | 17.40 |

S denotes the number of segregating sites (excluding indels) observed in European-American (EA) and African-American (AA) samples. Nucleotide diversity is denoted as π. The size (in bps) of each gene and the total amount sequenced are denoted as Seq. Len. and Seq. Amount, respectively.
