# Supplementary material for: Population History and Natural Selection Shape Patterns of Genetic Variation in 132 Genes
Source: PLoS Biol. 2004 Sep 7;2(10):e286. doi: 10.1371/journal.pbio.0020286 (PMC515367; doi:10.1371/journal.pbio.0020286)
Supplement: Table S2 — (534 KB DOC). [file pbio.0020286.st002.doc]

**Supplementary Table 2. Neutrality Test Statistics**

| Gene | Population | TD | p(TD) | D* | p(D*) | F* | p(F*) | H | p(H) |
| --- | --- | --- | --- | --- | --- | --- | --- | --- | --- |
| *IL1A** | EA | 2.437 | **0.0004** | 1.401 | 0.0150 | 2.092 | **0.0016** | 3.572 | 0.7964 |
|  | AA | 0.041 | 0.8818 | -0.213 | 0.7006 | -0.144 | 0.8690 | -1.033 | 0.3290 |
| *EPHB6** | EA | -2.022 | **0.0026** | -4.089 | **0.0008** | -3.999 | **0.0008** | -4.179 | 0.0964 |
|  | AA | -0.858 | 0.2426 | -0.354 | 0.5582 | -0.645 | 0.4144 | 2.548 | 0.5419 |
| *KEL** | EA | -2.209 | **0.0001** | -3.738 | **0.0022** | -3.807 | **0.0016** | -19.806 | **0.0002** |
|  | AA | -0.569 | 0.4314 | -0.596 | 0.3708 | -0.707 | 0.3482 | -7.215 | 0.1668 |
| *TRPV5** | EA | -2.066 | **0.0002** | -4.980 | **0.0001** | -4.691 | **0.0001** | -32.864 | **0.0001** |
|  | AA | -0.353 | 0.6068 | -0.620 | 0.3900 | -0.630 | 0.3896 | 4.815 | 0.5783 |
| *TRPV6** | EA | -2.735 | **0.0001** | -6.060 | **0.0001** | -5.797 | **0.0001** | -45.431 | **0.0001** |
|  | AA | 0.769 | 0.1958 | 0.768 | 0.2130 | 0.909 | 0.1440 | 1.814 | 0.4791 |
| *ABO** | EA | 2.337 | **0.0002** | 1.733 | **0.0001** | 2.320 | **0.0001** | -0.577 | 0.3717 |
|  | AA | 1.652 | 0.0126 | 1.417 | 0.0050 | 1.785 | 0.0026 | 2.827 | 0.4890 |
| *DCN** | EA | -1.988 | **0.0018** | -3.876 | **0.0006** | -3.827 | **0.0004** | -7.686 | **0.0150** |
|  | AA | -0.552 | 0.3976 | 0.599 | 0.3624 | 0.213 | 0.7648 | -4.278 | 0.2427 |
| *ACE2** | EA | 1.854 | **0.0058** | 1.626 | **0.0016** | 2.018 | **0.0024** | -2.569 | 0.1474 |
|  | AA | -0.439 | 0.4926 | 0.981 | 0.1110 | 0.547 | 0.4012 | 0.781 | 0.4635 |
| *APOH* | EA | 0.590 | 0.3420 | 0.459 | 0.4682 | 0.595 | 0.4000 | -6.233 | 0.1340 |
|  | AA | -0.690 | 0.3540 | -0.625 | 0.4296 | -0.774 | 0.3444 | -7.832 | 0.1729 |
| *BDKRB2* | EA | -0.428 | 0.6420 | -0.080 | 0.9602 | -0.250 | 0.7572 | -5.567 | 0.1150 |
|  | AA | -1.078 | 0.1614 | 0.829 | 0.1840 | 0.161 | 0.8510 | -9.289 | 0.0739 |
| *BF* | EA | -0.327 | 0.7880 | 0.817 | 0.1966 | 0.498 | 0.5596 | -2.613 | 0.1364 |
|  | AA | -0.489 | 0.6280 | -0.501 | 0.6520 | -0.596 | 0.5148 | 0.631 | 0.4518 |
| *C2* | EA | -0.630 | 0.4526 | 0.322 | 0.8244 | -0.021 | 0.9786 | -2.825 | 0.1466 |
|  | AA | -0.990 | 0.1880 | -1.945 | 0.0486 | -1.910 | 0.0500 | -1.533 | 0.2649 |
| *CCR2* | EA | 0.395 | 0.5676 | 0.956 | 0.3522 | 0.906 | 0.2806 | -1.170 | 0.2113 |
|  | AA | -1.013 | 0.2168 | -1.653 | 0.1112 | -1.696 | 0.0942 | -0.413 | 0.3356 |
| *CD36* | EA | 0.518 | 0.3774 | 0.467 | 0.4370 | 0.579 | 0.3994 | -1.694 | 0.3091 |
|  | AA | -0.815 | 0.2166 | -0.573 | 0.3700 | -0.791 | 0.2894 | -7.555 | 0.2019 |
| *CEBPB* | EA | 0.080 | 0.8166 | 0.603 | 0.8508 | 0.512 | 0.6238 | -3.046 | 0.0287 |
|  | AA | 0.033 | 0.8620 | -0.651 | 0.3380 | -0.501 | 0.6068 | 0.954 | 0.6399 |
| *CRF* | EA | 0.836 | 0.2852 | 0.771 | 0.2290 | 0.940 | 0.2524 | -1.429 | 0.2075 |
|  | AA | -0.496 | 0.6052 | -0.350 | 0.5642 | -0.484 | 0.5860 | -1.560 | 0.2396 |
| *CRP* | EA | 0.145 | 0.7688 | 0.953 | 0.4510 | 0.805 | 0.3818 | 1.506 | 0.9336 |
|  | AA | -0.277 | 0.8230 | -0.772 | 0.3216 | -0.712 | 0.4454 | 3.530 | 0.9782 |
| *CSF2* | EA | 0.111 | 0.7846 | -1.228 | 0.1510 | -0.925 | 0.3426 | 0.472 | 0.4578 |
|  | AA | -0.075 | 0.9594 | -0.068 | 0.7760 | -0.084 | 0.9330 | 2.320 | 0.8023 |
| *CSF3* | EA | 0.714 | 0.3596 | 1.210 | 0.2148 | 1.227 | 0.1490 | 1.297 | 0.6782 |
|  | AA | -0.746 | 0.4264 | -0.410 | 0.5362 | -0.632 | 0.5196 | 2.283 | 0.7566 |
| *CSF3R* | EA | -0.219 | 0.8484 | -1.881 | 0.0438 | -1.540 | 0.0988 | 1.087 | 0.4987 |
|  | AA | -0.704 | 0.3674 | 0.588 | 0.4424 | 0.130 | 0.8686 | -1.698 | 0.3087 |
| *CYP4A11* | EA | -0.903 | 0.2632 | 1.032 | 0.1570 | 0.407 | 0.5766 | -13.598 | **0.0068** |
|  | AA | -0.129 | 0.9350 | 0.454 | 0.5964 | 0.286 | 0.7192 | -1.715 | 0.2933 |
| *CYP4F2* | EA | 0.368 | 0.5434 | 1.352 | 0.0132 | 1.180 | 0.0784 | -6.766 | 0.1372 |
|  | AA | -0.433 | 0.5940 | -0.162 | 0.8046 | -0.320 | 0.6654 | -0.927 | 0.3598 |
| *F10* | EA | -0.137 | 0.8794 | -0.186 | 0.7032 | -0.209 | 0.7738 | 0.775 | 0.4585 |
|  | AA | -0.575 | 0.4124 | -1.385 | 0.1106 | -1.301 | 0.1270 | 3.286 | 0.6511 |
| *F11* | EA | 0.898 | 0.1560 | 0.402 | 0.5042 | 0.691 | 0.3244 | 2.176 | 0.5781 |
|  | AA | -0.426 | 0.5484 | 0.130 | 0.9276 | -0.095 | 0.8994 | 4.180 | 0.6049 |
| *F12* | EA | -0.372 | 0.7370 | -0.375 | 0.5236 | -0.450 | 0.6264 | -0.821 | 0.2577 |
|  | AA | -1.194 | 0.1344 | -0.939 | 0.2390 | -1.225 | 0.1904 | 3.019 | 0.7448 |
| *F13A1* | EA | 1.070 | 0.0810 | 1.206 | 0.0228 | 1.371 | 0.0244 | -7.832 | 0.1532 |
|  | AA | -0.244 | 0.7346 | -0.398 | 0.5030 | -0.415 | 0.5470 | -12.138 | 0.1371 |
| *F2* | EA | -0.889 | 0.2288 | 0.528 | 0.5430 | 0.025 | 0.9722 | -10.853 | **0.0168** |
|  | AA | -1.499 | 0.0330 | -0.701 | 0.3272 | -1.189 | 0.1634 | -9.840 | 0.0659 |
| *F2R* | EA | 0.252 | 0.6430 | -0.534 | 0.5308 | -0.311 | 0.6780 | 0.514 | 0.4511 |
|  | AA | -0.776 | 0.2768 | -1.103 | 0.1484 | -1.181 | 0.1506 | 3.878 | 0.7388 |
| *F2RL1* | EA | 0.680 | 0.3062 | -0.675 | 0.3338 | -0.232 | 0.7686 | 3.478 | 0.8239 |
|  | AA | -1.430 | 0.0424 | -2.768 | 0.0084 | -2.725 | 0.0074 | -6.114 | 0.1715 |
| *F2RL2* | EA | 0.943 | 0.2126 | 0.454 | 0.7020 | 0.743 | 0.3592 | 0.247 | 0.3829 |
|  | AA | -0.264 | 0.8250 | 0.585 | 0.5312 | 0.326 | 0.6822 | 4.820 | 0.8360 |
| *F2RL3* | EA | 0.411 | 0.5372 | -0.520 | 0.6522 | -0.244 | 0.7904 | 0.605 | 0.4678 |
|  | AA | -0.893 | 0.3040 | -1.046 | 0.2072 | -1.180 | 0.2132 | -3.241 | 0.1726 |
| *F3* | EA | 1.541 | 0.0474 | 0.506 | 0.6964 | 1.013 | 0.2052 | 0.352 | 0.4321 |
|  | AA | -0.831 | 0.2866 | -1.692 | 0.0912 | -1.646 | 0.0854 | -0.063 | 0.3748 |
| *F5* | EA | 0.406 | 0.4052 | 0.337 | 0.5588 | 0.424 | 0.4782 | -17.817 | 0.0735 |
|  | AA | -0.303 | 0.5784 | -0.698 | 0.2356 | -0.667 | 0.2730 | -24.672 | 0.0926 |
| *F7* | EA | -0.886 | 0.3044 | 0.706 | 0.2732 | 0.186 | 0.8340 | 0.180 | 0.3948 |
|  | AA | -1.156 | 0.1500 | -1.121 | 0.2442 | -1.354 | 0.1520 | 3.046 | 0.7722 |
| *F9* | EA | 1.005 | 0.1230 | 0.822 | 0.3154 | 1.054 | 0.1320 | -4.751 | 0.0883 |
|  | AA | -0.777 | 0.2192 | -0.812 | 0.2578 | -0.954 | 0.1898 | -15.336 | 0.0631 |
| *FGA* | EA | -0.609 | 0.5464 | -1.193 | 0.2920 | -1.186 | 0.2384 | 0.084 | 0.3774 |
|  | AA | -0.792 | 0.3800 | -0.520 | 0.6576 | -0.733 | 0.4404 | 0.317 | 0.4487 |
| *FGB* | EA | 0.563 | 0.4246 | 0.648 | 0.5182 | 0.727 | 0.3652 | -2.705 | 0.1778 |
|  | AA | -1.648 | 0.0252 | -0.534 | 0.4502 | -1.117 | 0.2324 | -7.910 | 0.0520 |
| *FGG* | EA | -0.516 | 0.6400 | -0.591 | 0.3766 | -0.667 | 0.5040 | 0.709 | 0.5840 |
|  | AA | -0.864 | 0.3366 | -1.389 | 0.1160 | -1.434 | 0.1536 | -0.894 | 0.2320 |
| *FGL2* | EA | 0.920 | 0.3220 | 1.110 | 0.5482 | 1.229 | 0.2006 | 0.299 | 0.4625 |
|  | AA | -1.059 | 0.2424 | -1.245 | 0.2804 | -1.405 | 0.1792 | -1.238 | 0.1927 |
| *FSBP* | EA | -0.562 | 0.5856 | -0.708 | 0.5096 | -0.780 | 0.4074 | 1.618 | 0.6517 |
|  | AA | -0.262 | 0.8384 | -0.193 | 0.9232 | -0.264 | 0.7696 | 1.711 | 0.6304 |
| *GP1BA* | EA | 0.027 | 0.8632 | -0.363 | 0.5342 | -0.279 | 0.7548 | 0.334 | 0.4141 |
|  | AA | -0.505 | 0.6390 | 1.475 | 0.0576 | 0.937 | 0.2454 | 1.525 | 0.6250 |
| *ICAM1* | EA | 0.036 | 0.8890 | -0.622 | 0.3826 | -0.473 | 0.5734 | 1.953 | 0.6884 |
|  | AA | -0.827 | 0.2750 | -1.782 | 0.0576 | -1.712 | 0.0728 | -0.615 | 0.3503 |
| *IFNG* | EA | 0.365 | 0.6076 | -1.154 | 0.1624 | -0.772 | 0.4384 | 0.320 | 0.4396 |
|  | AA | -1.374 | 0.0884 | -0.422 | 0.7166 | -0.897 | 0.3598 | -4.970 | 0.0713 |
| *IGF2* | EA | 1.076 | 0.2114 | 0.805 | 0.1814 | 1.051 | 0.2488 | -0.998 | 0.1812 |
|  | AA | -0.364 | 0.7732 | -0.021 | 0.8150 | -0.163 | 0.8730 | 2.792 | 0.9318 |
| *IGF2AS* | EA | 0.538 | 0.4002 | 0.424 | 0.6688 | 0.553 | 0.4646 | -4.625 | 0.1203 |
|  | AA | -0.172 | 0.8662 | -0.041 | 0.8628 | -0.105 | 0.8832 | 2.100 | 0.5368 |
| *IL10* | EA | 1.260 | 0.1192 | 0.506 | 0.7104 | 0.907 | 0.2820 | 2.879 | 0.9496 |
|  | AA | 0.784 | 0.3038 | 0.314 | 0.6094 | 0.566 | 0.5038 | 0.476 | 0.4393 |
| *IL10RA* | EA | -0.318 | 0.7206 | -0.400 | 0.6624 | -0.441 | 0.5902 | -16.097 | **0.0060** |
|  | AA | -0.339 | 0.6862 | -0.678 | 0.3308 | -0.661 | 0.4030 | 0.021 | 0.3945 |
| *IL10RB* | EA | 1.886 | **0.0020** | 0.197 | 0.8458 | 0.973 | 0.1156 | 0.361 | 0.4174 |
|  | AA | 0.142 | 0.7656 | -0.138 | 0.8110 | -0.041 | 0.9296 | -14.351 | 0.1094 |
| *IL11* | EA | 0.484 | 0.4784 | 0.131 | 0.9300 | 0.291 | 0.7432 | 1.150 | 0.5604 |
|  | AA | -0.216 | 0.8692 | 0.438 | 0.6762 | 0.239 | 0.7674 | 2.542 | 0.6594 |
| *IL12A* | EA | 1.160 | 0.1360 | 0.423 | 0.7660 | 0.806 | 0.3244 | -1.304 | 0.2358 |
|  | AA | -0.236 | 0.8384 | -0.632 | 0.3914 | -0.583 | 0.5084 | 3.476 | 0.7244 |
| *IL12B* | EA | 0.505 | 0.4402 | 0.322 | 0.6082 | 0.458 | 0.5862 | 3.996 | 0.9990 |
|  | AA | -0.132 | 0.9332 | -0.133 | 0.7430 | -0.165 | 0.8174 | 4.091 | 0.9060 |
| *IL13* | EA | -0.512 | 0.6280 | -0.929 | 0.4252 | -0.940 | 0.3420 | -4.106 | 0.0508 |
|  | AA | -0.202 | 0.9222 | 0.322 | 0.8714 | 0.167 | 0.8402 | 0.091 | 0.3765 |
| *IL15RA* | EA | 0.417 | 0.4498 | 0.292 | 0.6450 | 0.403 | 0.5450 | -0.862 | 0.3818 |
|  | AA | -0.598 | 0.3496 | -1.471 | 0.0716 | -1.378 | 0.0868 | 3.851 | 0.5385 |
| *IL17B* | EA | 1.030 | 0.1962 | 0.381 | 0.8452 | 0.715 | 0.4014 | -2.801 | 0.1176 |
|  | AA | -1.318 | 0.0966 | -3.374 | 0.0062 | -3.166 | 0.0068 | -4.847 | 0.0869 |
| *IL19* | EA | -0.509 | 0.6008 | -0.520 | 0.6622 | -0.612 | 0.5164 | -3.375 | 0.0966 |
|  | AA | -0.633 | 0.4800 | -1.593 | 0.1178 | -1.495 | 0.1278 | -0.582 | 0.3198 |
| *IL1B* | EA | -0.164 | 0.8884 | -1.104 | 0.1780 | -0.928 | 0.3074 | 0.741 | 0.4778 |
|  | AA | -0.397 | 0.6604 | -0.481 | 0.6080 | -0.544 | 0.5206 | 5.256 | 0.9787 |
| *IL1R1* | EA | 0.066 | 0.8642 | 0.347 | 0.7048 | 0.284 | 0.6852 | 7.484 | 0.8989 |
|  | AA | -1.024 | 0.1286 | -0.793 | 0.2512 | -1.050 | 0.1782 | 8.264 | 0.7875 |
| *IL1R2* | EA | 0.064 | 0.8552 | 0.998 | 0.0712 | 0.786 | 0.2364 | -19.618 | **0.0192** |
|  | AA | -0.472 | 0.5218 | -1.252 | 0.1138 | -1.149 | 0.1578 | -16.835 | 0.1049 |
| *IL1RN* | EA | 0.877 | 0.1884 | 1.265 | 0.0394 | 1.341 | 0.0378 | -19.945 | 0.0236 |
|  | AA | -1.123 | 0.1134 | -1.116 | 0.1650 | -1.343 | 0.1108 | -29.957 | 0.0185 |
| *IL2* | EA | 0.414 | 0.5642 | 0.716 | 0.2204 | 0.727 | 0.4478 | 1.211 | 0.8563 |
|  | AA | -1.557 | 0.0462 | -0.710 | 0.5666 | -1.186 | 0.2514 | 1.593 | 0.7443 |
| *IL20* | EA | -0.628 | 0.5454 | -1.193 | 0.2888 | -1.193 | 0.2358 | 1.397 | 0.8066 |
|  | AA | -0.641 | 0.5172 | -1.245 | 0.2566 | -1.236 | 0.2238 | 1.910 | 0.9166 |
| *IL21R* | EA | 0.291 | 0.6164 | -0.001 | 0.8920 | 0.124 | 0.8670 | -1.651 | 0.2769 |
|  | AA | -0.699 | 0.3278 | -0.912 | 0.2132 | -1.002 | 0.2058 | -0.496 | 0.3693 |
| *IL22* | EA | 0.878 | 0.2390 | 0.423 | 0.7498 | 0.690 | 0.4062 | -2.447 | 0.1640 |
|  | AA | -0.596 | 0.5288 | -0.853 | 0.3794 | -0.913 | 0.3368 | -1.323 | 0.2726 |
| *IL24* | EA | 0.888 | 0.2498 | -1.307 | 0.2332 | -0.668 | 0.4768 | -1.755 | 0.1095 |
|  | AA | -0.566 | 0.5554 | -0.775 | 0.4272 | -0.841 | 0.3652 | -6.600 | 0.0186 |
| *IL2RB* | EA | 0.664 | 0.2712 | -0.311 | 0.5846 | 0.051 | 0.9632 | -12.249 | 0.0668 |
|  | AA | 0.025 | 0.9202 | -0.143 | 0.8132 | -0.096 | 0.8664 | 1.156 | 0.4459 |
| *IL3* | EA | -0.132 | 0.9768 | -1.206 | 0.1434 | -1.018 | 0.3310 | -0.015 | 0.3419 |
|  | AA | -1.423 | 0.0828 | -1.645 | 0.0922 | -1.861 | 0.0852 | 0.144 | 0.3697 |
| *IL4* | EA | -0.458 | 0.5596 | 0.037 | 0.9084 | -0.173 | 0.8238 | -6.812 | 0.0855 |
|  | AA | -0.271 | 0.7312 | -0.179 | 0.8092 | -0.261 | 0.7160 | 3.403 | 0.5846 |
| *IL4R* | EA | 0.517 | 0.3726 | 1.217 | 0.0348 | 1.139 | 0.0700 | -13.876 | 0.0687 |
|  | AA | 0.127 | 0.7680 | 0.436 | 0.4892 | 0.376 | 0.5732 | -7.637 | 0.2122 |
| *IL5* | EA | -0.663 | 0.5868 | -1.660 | 0.3124 | -1.585 | 0.2398 | -0.725 | 0.0975 |
|  | AA | -1.044 | 0.2614 | -1.128 | 0.3412 | -1.300 | 0.2246 | 1.636 | 0.8329 |
| *IL6* | EA | 0.440 | 0.5168 | -0.109 | 0.7448 | 0.094 | 0.9104 | 0.574 | 0.4598 |
|  | AA | -1.015 | 0.2348 | -0.674 | 0.5084 | -0.945 | 0.3284 | -0.583 | 0.3089 |
| *IL8* | EA | 1.556 | 0.0950 | 0.608 | 0.8032 | 1.069 | 0.2400 | 0.233 | 0.4261 |
|  | AA | -1.444 | 0.0698 | -1.972 | 0.0532 | -2.129 | 0.0514 | -4.409 | 0.1029 |
| *IL9* | EA | -0.547 | 0.6208 | -0.093 | 0.6818 | -0.285 | 0.7608 | -2.256 | 0.0704 |
|  | AA | -1.083 | 0.2172 | -0.488 | 0.6736 | -0.825 | 0.3906 | 0.417 | 0.4210 |
| *IL9R* | EA | -0.307 | 0.7650 | -0.161 | 0.8614 | -0.261 | 0.7426 | -7.282 | 0.0620 |
|  | AA | 0.067 | 0.8512 | -0.392 | 0.5574 | -0.267 | 0.7456 | 2.829 | 0.5596 |
| *IRAK4* | EA | -1.414 | 0.0304 | -0.664 | 0.3324 | -1.119 | 0.1736 | -23.674 | **0.0014** |
|  | AA | -0.991 | 0.1314 | -1.591 | 0.0530 | -1.636 | 0.0488 | 0.149 | 0.4059 |
| *ITGA2* | EA | 0.406 | 0.3650 | 0.842 | 0.0776 | 0.805 | 0.1202 | -2.051 | 0.3795 |
|  | AA | 0.307 | 0.4832 | 0.554 | 0.2906 | 0.552 | 0.2890 | 14.194 | 0.7770 |
| *ITGA8* | EA | -0.118 | 0.8686 | 0.665 | 0.2976 | 0.452 | 0.4764 | -22.404 | **0.0130** |
|  | AA | -0.537 | 0.3322 | -1.428 | 0.0568 | -1.318 | 0.0782 | -8.945 | 0.2169 |
| *JAK3* | EA | 0.529 | 0.4014 | 0.416 | 0.6554 | 0.543 | 0.4592 | -6.080 | 0.0858 |
|  | AA | -0.267 | 0.7554 | 0.105 | 0.9766 | -0.044 | 0.9432 | -16.441 | 0.0371 |
| *KLK1* | EA | 0.582 | 0.4000 | -0.393 | 0.6980 | -0.059 | 0.9306 | -2.766 | 0.1933 |
|  | AA | -0.435 | 0.6550 | 0.353 | 0.6030 | 0.085 | 0.9188 | -3.910 | 0.1820 |
| *KLKB1* | EA | 1.522 | 0.0144 | 0.626 | 0.3638 | 1.139 | 0.0678 | 6.792 | 0.8155 |
|  | AA | 0.232 | 0.6442 | 0.458 | 0.4368 | 0.448 | 0.4818 | 9.535 | 0.8537 |
| *KNG* | EA | 1.523 | 0.0190 | 1.247 | 0.0200 | 1.599 | 0.0082 | -14.029 | 0.1063 |
|  | AA | 1.042 | 0.0846 | 0.085 | 0.9028 | 0.528 | 0.4066 | -6.371 | 0.2469 |
| *LTA* | EA | 1.063 | 0.1884 | 0.174 | 0.9582 | 0.555 | 0.5178 | 1.594 | 0.7038 |
|  | AA | 0.309 | 0.6312 | 0.905 | 0.4130 | 0.831 | 0.3536 | 0.938 | 0.5224 |
| *LTB* | EA | -1.202 | 0.1994 | -1.002 | 0.4994 | -1.248 | 0.2420 | -1.133 | 0.1286 |
|  | AA | -1.590 | 0.0512 | -0.230 | 0.5940 | -0.816 | 0.4198 | -0.115 | 0.3212 |
| *MAP3K8* | EA | 0.819 | 0.1866 | -0.123 | 0.7532 | 0.246 | 0.7498 | -4.844 | 0.1241 |
|  | AA | -0.818 | 0.2106 | -0.412 | 0.4938 | -0.669 | 0.3560 | -9.989 | 0.1378 |
| *MC1R* | EA | 0.676 | 0.3642 | 0.314 | 0.5988 | 0.519 | 0.5556 | -4.449 | 0.0392 |
|  | AA | -0.240 | 0.8798 | -0.654 | 0.5272 | -0.603 | 0.5194 | 0.511 | 0.4177 |
| *MMP3* | EA | 0.331 | 0.6090 | 0.424 | 0.5040 | 0.465 | 0.5790 | -5.267 | 0.0763 |
|  | AA | -0.221 | 0.8668 | -0.222 | 0.6554 | -0.271 | 0.7310 | 2.516 | 0.6290 |
| *MMP9* | EA | 0.587 | 0.4054 | 0.314 | 0.5964 | 0.477 | 0.5850 | 0.826 | 0.4839 |
|  | AA | -0.196 | 0.8824 | -0.295 | 0.7728 | -0.309 | 0.7066 | 2.628 | 0.6470 |
| *NOS3* | EA | 0.793 | 0.2256 | 0.486 | 0.4262 | 0.700 | 0.3342 | -1.288 | 0.2893 |
|  | AA | -0.739 | 0.2846 | -0.960 | 0.2042 | -1.049 | 0.1960 | -2.344 | 0.2840 |
| *PFC* | EA | 0.593 | 0.4508 | 0.956 | 0.4394 | 0.980 | 0.2660 | -3.637 | 0.0357 |
|  | AA | -0.263 | 0.8590 | -0.358 | 0.5400 | -0.392 | 0.6714 | 1.653 | 0.7075 |
| *PLAT* | EA | -0.326 | 0.6656 | 0.027 | 0.9470 | -0.120 | 0.8494 | -9.287 | 0.0575 |
|  | AA | -1.175 | 0.0666 | -1.251 | 0.1118 | -1.467 | 0.0726 | -13.505 | 0.1201 |
| *PLAU* | EA | 0.807 | 0.3022 | -1.064 | 0.1964 | -0.511 | 0.5810 | 1.368 | 0.6431 |
|  | AA | 0.474 | 0.4806 | -0.715 | 0.4972 | -0.353 | 0.6958 | 3.265 | 0.9390 |
| *PLAUR* | EA | -0.573 | 0.4382 | -0.034 | 0.9848 | -0.281 | 0.7182 | -20.846 | **0.0141** |
|  | AA | -0.836 | 0.2300 | -0.232 | 0.6586 | -0.551 | 0.4636 | -22.536 | 0.0496 |
| *PLG* | EA | 1.407 | 0.0262 | 0.248 | 0.6976 | 0.801 | 0.2262 | -3.637 | 0.2501 |
|  | AA | 0.301 | 0.5746 | 0.255 | 0.7086 | 0.325 | 0.6458 | -3.381 | 0.3005 |
| *PON1* | EA | -0.200 | 0.8098 | -0.485 | 0.4308 | -0.462 | 0.5132 | -20.390 | 0.0299 |
|  | AA | 0.107 | 0.7994 | -0.003 | 0.9254 | 0.037 | 0.9698 | 9.135 | 0.7585 |
| *PON2* | EA | 0.915 | 0.1240 | 1.166 | 0.0296 | 1.273 | 0.0396 | -2.898 | 0.2612 |
|  | AA | -0.216 | 0.7808 | -0.912 | 0.2278 | -0.783 | 0.2900 | 0.683 | 0.4345 |
| *PPARA* | EA | -0.769 | 0.2570 | -0.864 | 0.2714 | -0.990 | 0.2028 | -13.921 | **0.0105** |
|  | AA | -0.821 | 0.1876 | -1.019 | 0.1814 | -1.130 | 0.1446 | -2.059 | 0.3181 |
| *PPARG* | EA | 0.576 | 0.3026 | -0.557 | 0.3804 | -0.174 | 0.7802 | -2.442 | 0.2811 |
|  | AA | -0.148 | 0.8462 | -0.087 | 0.9076 | -0.138 | 0.8282 | 3.125 | 0.5647 |
| *PROC* | EA | 1.565 | 0.0414 | 0.406 | 0.5272 | 0.967 | 0.2116 | 1.586 | 0.5501 |
|  | AA | -0.036 | 0.9490 | -0.339 | 0.5846 | -0.282 | 0.7436 | 0.629 | 0.4292 |
| *PROCR* | EA | 0.648 | 0.4122 | -0.958 | 0.2166 | -0.505 | 0.6138 | 0.442 | 0.4787 |
|  | AA | 0.132 | 0.7820 | 0.129 | 0.7276 | 0.147 | 0.8904 | -0.394 | 0.2974 |
| *PROS1* | EA | -0.143 | 0.8758 | -0.792 | 0.3482 | -0.677 | 0.3772 | 3.480 | 0.9243 |
|  | AA | -1.632 | 0.0050 | -1.847 | 0.0208 | -2.115 | 0.0116 | 5.733 | 0.9403 |
| *PROZ* | EA | 0.249 | 0.6646 | -0.858 | 0.3654 | -0.555 | 0.5302 | -0.757 | 0.3104 |
|  | AA | -1.397 | 0.0512 | -1.835 | 0.0532 | -1.999 | 0.0462 | -4.860 | 0.1910 |
| *PTGS2* | EA | -0.794 | 0.3524 | -1.028 | 0.3052 | -1.130 | 0.2316 | -3.662 | 0.0705 |
|  | AA | -0.854 | 0.2992 | -1.364 | 0.1156 | -1.407 | 0.1340 | -1.807 | 0.2336 |
| *SCYA2* | EA | 0.203 | 0.7190 | 0.813 | 0.2142 | 0.709 | 0.4084 | 0.807 | 0.4900 |
|  | AA | -0.791 | 0.3662 | -0.723 | 0.4622 | -0.889 | 0.3462 | 4.368 | 0.9721 |
| *SELE* | EA | -0.570 | 0.4898 | 0.281 | 0.8106 | -0.028 | 0.9726 | -11.485 | 0.0508 |
|  | AA | -0.792 | 0.3214 | -1.539 | 0.0846 | -1.518 | 0.1044 | -3.549 | 0.2396 |
| *SELL* | EA | -0.504 | 0.4962 | -0.072 | 0.8214 | -0.281 | 0.6944 | -9.990 | 0.0997 |
|  | AA | -0.378 | 0.6132 | -1.301 | 0.1022 | -1.146 | 0.1556 | 5.292 | 0.5960 |
| *SELP* | EA | 0.290 | 0.5612 | 0.413 | 0.4782 | 0.439 | 0.4828 | -25.211 | **0.0189** |
|  | AA | -0.814 | 0.1690 | -1.207 | 0.0978 | -1.268 | 0.0872 | -4.348 | 0.3135 |
| *SELPLG* | EA | 0.162 | 0.7618 | -0.842 | 0.3518 | -0.579 | 0.4938 | -7.265 | 0.0674 |
|  | AA | -0.679 | 0.3790 | -1.231 | 0.1404 | -1.229 | 0.1676 | -3.675 | 0.2180 |
| *SERPINA5* | EA | 1.787 | 0.0240 | 1.487 | 0.0346 | 1.895 | 0.0104 | -1.664 | 0.2432 |
|  | AA | 0.179 | 0.7142 | -0.102 | 0.7942 | -0.009 | 0.9882 | -0.711 | 0.3214 |
| *SERPINC1* | EA | -0.680 | 0.4214 | -0.543 | 0.4286 | -0.700 | 0.4570 | -5.372 | 0.0479 |
|  | AA | -0.181 | 0.8978 | 0.015 | 0.8526 | -0.072 | 0.9044 | -1.117 | 0.2803 |
| *SERPINE1* | EA | 0.689 | 0.3214 | 0.547 | 0.4044 | 0.711 | 0.3636 | 1.228 | 0.5030 |
|  | AA | -0.754 | 0.3640 | -1.505 | 0.1146 | -1.478 | 0.1118 | -0.242 | 0.3582 |
| *SFTPA1* | EA | -1.347 | 0.0498 | -0.603 | 0.4134 | -1.047 | 0.1994 | -38.017 | **0.0097** |
|  | AA | -0.392 | 0.5902 | -1.017 | 0.1910 | -0.947 | 0.2204 | -10.430 | 0.1727 |
| *SFTPA2* | EA | -1.452 | 0.0402 | -0.695 | 0.3264 | -1.164 | 0.1678 | -26.808 | **0.0132** |
|  | AA | -0.444 | 0.5758 | -0.332 | 0.6430 | -0.453 | 0.5492 | -10.948 | 0.1498 |
| *SFTPB* | EA | 0.909 | 0.2544 | 0.750 | 0.2406 | 0.941 | 0.2736 | 0.430 | 0.4769 |
|  | AA | -0.099 | 0.9902 | -0.580 | 0.4120 | -0.494 | 0.5678 | 1.414 | 0.5329 |
| *SFTPC* | EA | 0.577 | 0.4276 | 1.037 | 0.2308 | 1.033 | 0.1896 | -0.355 | 0.3244 |
|  | AA | -0.547 | 0.5712 | -1.612 | 0.0950 | -1.476 | 0.1460 | -0.093 | 0.3545 |
| *SFTPD* | EA | 0.106 | 0.8036 | -0.090 | 0.8966 | -0.030 | 0.9636 | 0.969 | 0.4330 |
|  | AA | -0.606 | 0.3906 | -0.408 | 0.5136 | -0.582 | 0.4472 | -8.975 | 0.1580 |
| *SMP1* | EA | -0.085 | 0.9748 | -0.922 | 0.2262 | -0.748 | 0.3834 | 1.732 | 0.6699 |
|  | AA | -0.456 | 0.5570 | -1.322 | 0.1078 | -1.200 | 0.1542 | -1.597 | 0.2964 |
| *STAT4* | EA | -0.637 | 0.3918 | -1.583 | 0.0598 | -1.483 | 0.0850 | -2.614 | 0.2071 |
|  | AA | -1.249 | 0.0634 | -1.584 | 0.0694 | -1.744 | 0.0464 | 0.875 | 0.4439 |
| *STAT6* | EA | 0.389 | 0.5652 | -0.705 | 0.3124 | -0.401 | 0.6336 | -2.419 | 0.1264 |
|  | AA | -1.112 | 0.1356 | -0.734 | 0.4038 | -1.035 | 0.2318 | -2.276 | 0.2296 |
| *TF* | EA | 0.980 | 0.0846 | 0.107 | 0.8548 | 0.499 | 0.4432 | -9.977 | 0.1247 |
|  | AA | -0.512 | 0.4300 | 0.072 | 0.9308 | -0.179 | 0.7700 | -20.999 | 0.0626 |
| *TFPI* | EA | 0.176 | 0.7134 | 0.887 | 0.1834 | 0.751 | 0.2528 | 3.414 | 0.6818 |
|  | AA | -1.122 | 0.0698 | -0.688 | 0.2830 | -1.015 | 0.1692 | -16.016 | 0.0939 |
| *TGFB3* | EA | -0.145 | 0.8776 | -0.581 | 0.4944 | -0.517 | 0.5108 | -8.979 | 0.0396 |
|  | AA | -0.425 | 0.5800 | -0.939 | 0.2656 | -0.897 | 0.2752 | 0.150 | 0.4040 |
| *THBD* | EA | -1.053 | 0.2374 | -2.163 | 0.0730 | -2.127 | 0.0570 | 0.900 | 0.6536 |
|  | AA | -1.686 | 0.0296 | -2.972 | 0.0180 | -3.005 | 0.0136 | 1.700 | 0.7524 |
| *TIRAP* | EA | 0.401 | 0.4966 | 1.249 | 0.0622 | 1.126 | 0.1058 | 5.212 | 0.9537 |
|  | AA | -0.959 | 0.1910 | -0.596 | 0.3950 | -0.875 | 0.2942 | 3.293 | 0.6920 |
| *TNF* | EA | -0.901 | 0.3630 | 1.441 | 0.1620 | 0.792 | 0.4044 | -0.880 | 0.1700 |
|  | AA | -1.703 | 0.0278 | -1.082 | 0.3356 | -1.534 | 0.1436 | -0.028 | 0.3507 |
| *TNFAIP1* | EA | -0.449 | 0.6486 | 0.330 | 0.9342 | 0.083 | 0.9436 | 0.937 | 0.7075 |
|  | AA | -1.639 | 0.0252 | -0.699 | 0.4330 | -1.236 | 0.1742 | -6.502 | 0.0541 |
| *TNFAIP2* | EA | -0.129 | 0.9496 | -1.576 | 0.1026 | -1.277 | 0.1626 | -5.693 | 0.0877 |
|  | AA | -0.322 | 0.7278 | -0.318 | 0.6132 | -0.388 | 0.6342 | -2.059 | 0.2589 |
| *TNFAIP3* | EA | -0.497 | 0.5664 | -1.327 | 0.1836 | -1.232 | 0.1880 | -5.257 | 0.0561 |
|  | AA | -0.103 | 0.9602 | -0.861 | 0.3396 | -0.704 | 0.4138 | 2.329 | 0.6462 |
| *TNFRSF1A* | EA | 0.974 | 0.1886 | 0.442 | 0.7642 | 0.741 | 0.3670 | 0.336 | 0.4301 |
|  | AA | -0.533 | 0.5342 | -0.604 | 0.4980 | -0.696 | 0.4120 | 3.547 | 0.6957 |
| *TNFRSF1B* | EA | -0.517 | 0.3806 | 0.425 | 0.5636 | 0.100 | 0.9028 | -30.683 | **0.0026** |
|  | AA | -1.141 | 0.0588 | -0.580 | 0.3852 | -0.943 | 0.1796 | -30.915 | 0.0086 |
| *TRAF6* | EA | -1.075 | 0.1336 | -1.407 | 0.1208 | -1.534 | 0.0864 | -5.859 | 0.0930 |
|  | AA | -1.170 | 0.0898 | -0.771 | 0.2870 | -1.099 | 0.1900 | 3.341 | 0.5936 |
| *VCAM1* | EA | 0.118 | 0.8316 | -0.235 | 0.6458 | -0.139 | 0.8574 | -3.571 | 0.1294 |
|  | AA | -1.223 | 0.0788 | -1.236 | 0.1186 | -1.476 | 0.0878 | -7.318 | 0.1446 |
| *VEGF* | EA | 0.717 | 0.3000 | 0.106 | 0.9828 | 0.375 | 0.6328 | 0.552 | 0.4351 |
|  | AA | -0.396 | 0.6536 | -1.127 | 0.2246 | -1.037 | 0.2474 | -0.410 | 0.3490 |
| *VTN* | EA | -0.433 | 0.7324 | 1.118 | 0.0738 | 0.707 | 0.4494 | -8.295 | **0.0032** |
|  | AA | -0.220 | 0.9130 | 0.652 | 0.3324 | 0.415 | 0.6316 | -6.014 | 0.0558 |

D, D*, F*, and H denote Tajima’s D, Fu and Li’s D*, Fu and Li’s F*, and Fay and Wu’s H, respectively. Demographically robust selection genes are indicated with *. P-values were determined by 10^4^ coalescent simulations with recombination assuming a standard neutral model as described in the text. P-values that remained significant (FDR = 5%) after correcting for multiple tests are shown in bold.
