# Supplementary material for: Population History and Natural Selection Shape Patterns of Genetic Variation in 132 Genes
Source: PLoS Biol. 2004 Sep 7;2(10):e286. doi: 10.1371/journal.pbio.0020286 (PMC515367; doi:10.1371/journal.pbio.0020286)
Supplement: Table S3 — (87 KB DOC). [file pbio.0020286.st003.doc]

**Supplementary Table 3. Overlap of Genes Analyzed by Clark et al. (2003)**

| **Genes** | **LocusLink Id** | **Model 2 p-value human** | **Model 2 p-value chimp** | **dN/dS human** | **dN/dS chimp** |
| --- | --- | --- | --- | --- | --- |
| *TRPV6** | 55503 | **0.0002** | 1.0000 | 280.0000 | 0.1160 |
| *ICAM1* | 3383 | **0.0027** | 1.0000 | 0.7700 | 458.0000 |
| *SFTPC* | 6440 | **0.0124** | 0.2170 | 48.7000 | 0.0001 |
| *PFC* | 5199 | **0.0150** | 0.3930 | 0.7800 | 0.1640 |
| *IL15RA* | 3601 | **0.0181** | 0.1110 | 115.0000 | 0.6280 |
| *EPHB6** | 2051 | **0.0353** | 0.4410 | 1.0300 | 0.1730 |
| *SMP1* | 23585 | **0.0382** | 1.0000 | 0.9670 | 0.6410 |
| *SERPINE1* | 5054 | **0.0428** | 1.0000 | 1000.0000 | 47.2000 |
| *PPARG* | 5468 | **0.0452** | **0.0191** | 0.1630 | 0.0001 |
| *DCN** | 1634 | 1.0000 | **0.0034** | 0.0001 | 0.0001 |
| *KLKB1* | 3818 | 0.1010 | 0.4340 | 1.0900 | 0.3400 |
| *IRAK4* | 51135 | 0.1400 | 0.3610 | 1000.0000 | 1000.0000 |
| *VTN* | 7448 | 0.1480 | 0.1920 | 0.3050 | 0.0001 |
| *IL1RN* | 3557 | 0.1570 | 0.4350 | 77.8000 | 0.0001 |
| *IL19* | 29949 | 0.1710 | 0.4340 | 73.6000 | 0.0001 |
| *IL1R1* | 3554 | 0.1850 | 0.3540 | 0.3650 | 0.0001 |
| *PON1* | 5444 | 0.2210 | **0.0018** | 0.2810 | 0.2350 |
| *MAP3K8* | 1326 | 0.2410 | 0.3980 | 0.1400 | 0.0001 |
| *F11* | 2160 | 0.2610 | 0.3990 | 0.2670 | 0.0800 |
| *IL1B* | 3553 | 0.2700 | 0.1820 | 0.3270 | 16.0000 |
| *F2RL1* | 2150 | 0.3440 | 0.1720 | 0.0001 | 29.9000 |
| *FSBP* | 10646 | 0.3500 | **0.0242** | 1000.0000 | 1000.0000 |
| *CCR2* | 1231 | 0.3720 | 1.0000 | 13.1000 | 0.0001 |
| *TNFAIP3* | 7126 | 0.3870 | **0.0279** | 0.0001 | 17.0000 |
| *CSF3R* | 1441 | 0.3960 | 0.4340 | 0.3970 | 0.1710 |
| *VCAM1* | 7412 | 0.4320 | 0.1350 | 0.2570 | 0.4580 |
| *IL17B* | 27190 | 0.4470 | 0.3880 | 0.0001 | 0.0001 |
| *CRP* | 1401 | 0.4500 | 1.0000 | 0.0001 | 67.9000 |
| *APOH* | 350 | 1.0000 | 0.4260 | 0.0001 | 0.0001 |
| *C2* | 717 | 1.0000 | 1.0000 | 0.0001 | 0.2530 |
| *CSF3* | 1440 | 1.0000 | 1.0000 | 0.0001 | 0.0001 |
| *F2RL2* | 2151 | 1.0000 | 1.0000 | 0.0001 | 0.0001 |
| *F9* | 2158 | 1.0000 | 0.4850 | 0.0001 | 12.5000 |
| *FGB* | 2244 | 1.0000 | 1.0000 | 0.0001 | 0.3510 |
| *FGG* | 2266 | 1.0000 | 1.0000 | 0.1190 | 0.0771 |
| *IGF2* | 3481 | 1.0000 | 1.0000 | 0.0001 | 0.0001 |
| *IL10* | 3586 | 1.0000 | 0.0546 | 0.0001 | 0.3400 |
| *IL20* | 50604 | 1.0000 | 0.1130 | 0.0001 | 0.0001 |
| *IL21R* | 50615 | 1.0000 | 0.4460 | 0.2130 | 0.1070 |
| *IL2RB* | 3560 | 1.0000 | 1.0000 | 34.6000 | 0.0765 |
| *IL5* | 3567 | 1.0000 | 0.2880 | 0.0001 | 0.0001 |
| *PON2* | 5445 | 1.0000 | 0.4430 | 0.0001 | 0.0001 |
| *SELL* | 6402 | 1.0000 | 0.4310 | 0.0001 | 0.0001 |
| *SERPINC1* | 462 | 1.0000 | 0.2210 | 0.0001 | 0.1740 |
| *STAT4* | 6775 | 1.0000 | 1.0000 | 0.0001 | 0.0001 |
| *STAT6* | 6778 | 1.0000 | 0.4450 | 0.0001 | 1.6500 |
| *TGFB3* | 7043 | 1.0000 | 0.4260 | 0.0001 | 0.0001 |
| *TNF* | 7124 | 1.0000 | 1.0000 | 0.0001 | 0.0001 |
| *TNFRSF1B* | 7133 | 1.0000 | 0.2530 | 0.2330 | 0.0576 |
| *VEGF* | 7422 | 1.0000 | 0.4610 | 0.0001 | 0.0001 |

Summary of the 50 overlapping genes analyzed by Clark et al (2003). The data was downloaded from <https://panther.appliedbiosystems.com/appleraHCM_alignments/index.jsp> [DatabaseS2]. Model 2 p-value human and chimp denote the significance of accelerated evolution along the human and chimp lineage for each gene using a codon based maximum likelihood test. Nominally significant P-values (P < 0.05) are shown in bold. Demographically robust selection genes (see text for details) are denoted by a *.
